# Ethanolamine metabolism through two genetically distinct loci enables *Klebsiella pneumoniae* to bypass nutritional competition in the gut

**DOI:** 10.1371/journal.ppat.1012189

**Published:** 2024-05-07

**Authors:** Andrew J. Barnes, Emma F. Bennett, Ben Vezina, Andrew W. Hudson, Giovanna E. Hernandez, Noah A. Nutter, Andrew S. Bray, Ravinder Nagpal, Kelly L. Wyres, M. Ammar Zafar

**Affiliations:** 1 Department of Microbiology and Immunology, Wake Forest School of Medicine, Winston-Salem, North Carolina, United States of America; 2 Department of Infectious Diseases, Central Clinical School, Monash University, Melbourne, Victoria, Australia; 3 Department of Health, Nutrition, and Food Science, Florida State University, Tallahassee, Florida, United States of America; Tufts University, UNITED STATES

## Abstract

Successful microbial colonization of the gastrointestinal (GI) tract hinges on an organism’s ability to overcome the intense competition for nutrients in the gut between the host and the resident gut microbiome. Enteric pathogens can exploit ethanolamine (EA) in the gut to bypass nutrient competition. However, *Klebsiella pneumoniae* (*K*. *pneumoniae*) is an asymptomatic gut colonizer and, unlike well-studied enteric pathogens, harbors two genetically distinct ethanolamine utilization (*eut*) loci. Our investigation uncovered unique roles for each *eut* locus depending on EA utilization as a carbon or nitrogen source. Murine gut colonization studies demonstrated the necessity of both *eut* loci in the presence of intact gut microbiota for robust GI colonization by *K*. *pneumoniae*. Additionally, while some *Escherichia coli* gut isolates could metabolize EA, other commensals were incapable, suggesting that EA metabolism likely provides *K*. *pneumoniae* a selective advantage in gut colonization. Molecular and bioinformatic analyses unveiled the conservation of two *eut* loci among *K*. *pneumoniae* and a subset of the related taxa in the *K*. *pneumoniae* species complex, with the NtrC-RpoN regulatory cascade playing a pivotal role in regulation. These findings identify EA metabolism as a critical driver of *K*. *pneumoniae* niche establishment in the gut and propose microbial metabolism as a potential therapeutic avenue to combat *K*. *pneumoniae* infections.

## Author summary

*Klebsiella pneumoniae* (*K*. *pneumoniae*) is a leading cause of nosocomial infections but is considered a silent gut colonizer. A molecular understanding of how *K*. *pneumoniae* colonizes the GI tract and overcomes host- and resident gut microbiota-mediated colonization resistance requires further investigation. A direct mechanism of colonization resistance is through competition for the same nutrient resources. *K*. *pneumoniae* can sidestep nutrient competition by metabolizing ethanolamine as an alternative nutrient source, thus overcoming microbiota-mediated colonization resistance. The unique ethanolamine utilization loci of *K*. *pneumoniae* are both required for robust gut colonization and are conserved among *K*. *pneumoniae*, suggesting that ethanolamine metabolism is an important mediator of gut colonization.

## Introduction

The gastrointestinal (GI) tract is the most abundant site of bacterial colonization within humans, harboring ~10^14^ cells [1]. The resident commensals of the GI tract continuously compete with each other and the host for nutrients [2]. This constant battle for energy sources consequentially leads to a protective phenomenon known as colonization resistance (CR), as incoming microorganisms, including pathogens, are prevented from establishing themselves in the GI tract due to limited nutrient availability [3,4]. The gut microbiota can employ various tactics to enhance CR, including toxin production, host-response modulation, pH modification, and nutrient competition [5]. One mechanism that can allow an incoming microorganism to overcome CR in the GI tract is increased metabolic capacity, which maximizes the ability to adapt and persist under fluctuating nutrient availability [6].

*Klebsiella pneumoniae* (*K*. *pneumoniae*) is considered a priority pathogen by the Centers for Disease Control and Prevention due to its prevalence in healthcare-associated infections (HAIs) and multi-drug resistance [7,8]. Recent studies have demonstrated that *K*. *pneumoniae* asymptomatically colonizes the GI tract before translocating to sterile sites within the host and transmitting via the fecal-oral route [9,10]. This initial asymptomatic GI colonization groups *K*. *pneumoniae* with opportunistic pathogens (pathobiont) as it can reside in the gut silently until microflora-disrupting events such as antibiotic treatment or immune suppression, lead to *Klebsiella*-associated disease states [11–14]. However, there are significant gaps in our knowledge of the factors contributing to the initial asymptomatic GI colonization.

The GI tract contains a diverse selection of nutrient sources, which are constantly sought after by the resident gut microbiome and the host. Incoming *K*. *pneumoniae* must have the metabolic capacity to compete with established commensals in the dynamic gut milieu. *K*. *pneumoniae* possesses genes that encode for a diverse set of carbon and nitrogen metabolism pathways [15], and we recently demonstrated that it could overcome CR by metabolizing fucose as an alternative carbon source in the gut [16].

**Fig 1:**
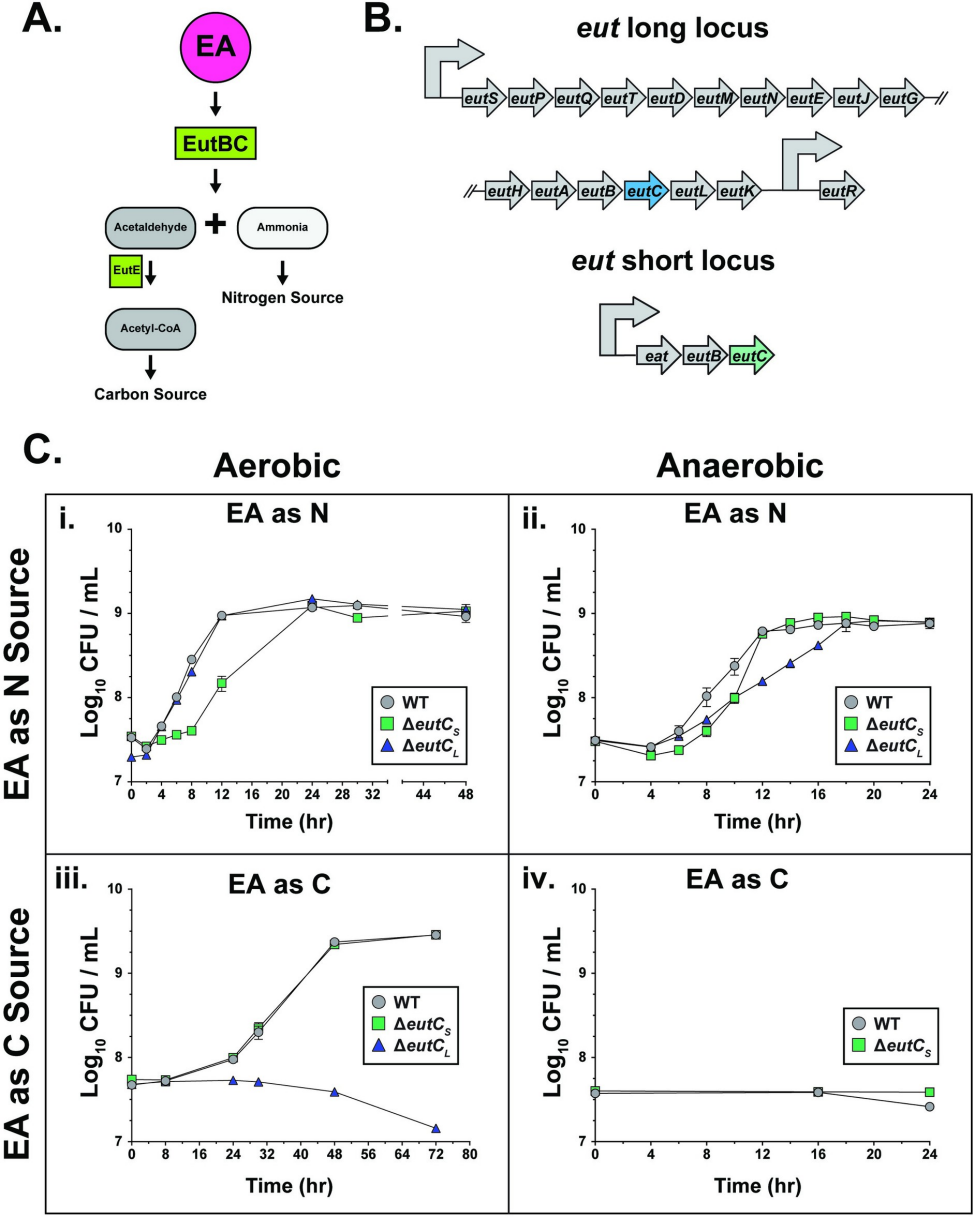
*K*. *pneumoniae* ethanolamine utilization (*eut*) loci provide growth advantages when EA is provided as a carbon or nitrogen source. **(A)** Graphic representation of ethanolamine (EA) breakdown via the EutBC ammonia lyase into respective carbon and nitrogen sources. Enzymes are highlighted in green **(B)** Genetic organization of the *eut* long- and short-locus of *K*. *pneumoniae* isolate KPPR1S with the deleted genes highlighted. **(C)** *In vitro* aerobic (**i** & **iii**) and anaerobic (**ii & iv**) growth kinetics as depicted by CFU/mL over time of the wild-type (WT) *K*. *pneumoniae* or single locus mutants (*ΔeutC*_*s*_ & *ΔeutC*_*L*_) grown with EA as the sole nitrogen (**i & ii**) or the sole carbon (**iii & iv**) source at 37°C. Strains were grown in M9 minimal media (MM) supplemented with 0.4% Gly and 2.5 mM EA (**i** & **ii**) or 20 mM EA and 10 mM NH_4_Cl (**iii** & **iv**). All strains grown with EA as a nutrient source included 200nM B12. Mean ± SEM for ≥ 3 independent experiments is shown. N = nitrogen; C = carbon.

Phospholipid phosphatidylethanolamine (PE), a dominant membrane component of both prokaryotic and eukaryotic cells, is abundant within the GI tract and is continuously modified and recycled for alternative uses [17,18]. Bacterial phosphodiesterases hydrolyze PE into glycerol and ethanolamine (EA) [19], which can be utilized by a variety of bacteria, including but not limited to *Salmonella sp*., *Escherichia coli* (*E*. *coli*) and *Pseudomonas aeruginosa* (*P*. *aeruginosa*) [20]. These bacteria contain specialized ethanolamine utilization (*eut*) genes, which encode proteins that facilitate the conversion of EA into ammonia and acetaldehyde as nitrogen and carbon sources, respectively [21]. Ammonia can be used to synthesize amino acids like glycine and glutamate, while acetaldehyde can be converted to ethanol or acetyl-CoA for use in the TCA cycle or fatty acid biosynthesis **(Fig 1A)**. There are two distinct *eut* loci: 1) A long locus encompassing ~16 genes that encode proteins that facilitate EA uptake, utilization, and locus regulation, and 2) A short locus that only contains genes responsible for importing and metabolizing EA. Both loci contain *eutB* and *eutC*, which encode for the ethanolamine ammonia-lyase (EutBC) **(Fig 1B)**. It has been established that *Firmicutes*, commensal and pathogenic *E*. *coli* strains and *Salmonella sp*. can metabolize EA via the long *eut* locus [22–24], while *P*. *aeruginosa* and *Acinetobacter baumannii* (*A*. *baumannii*) possess the short locus [25,26]. EutR in *Enterobacteriaceae* and the EutVW two-component system of *Firmicutes*, have been implicated in regulating the long locus. Conversely, the short locus expression is regulated by the enhancer protein EatR in conjunction with the alternative sigma factor (RpoN; σ^54^) [23]. While most bacteria contain only one *eut* locus, *K*. *pneumoniae* is unique because it possesses both a short and a long *eut* locus [20]. As there can be a metabolic cost associated with the synthesis of enzymes, we speculated whether both loci were functional, if there was a biological cost associated with retaining two distinct *eut* loci, and whether their functions were unique or redundant. Furthermore, while the utilization of EA and regulation of the *eut* locus has been investigated in other bacterial species, primarily those that cause inflammation in the GI tract [27–31], there is a lack of understanding regarding EA metabolism and regulation of the *eut* loci within *K*. *pneumoniae*.

Herein, we identified the contribution of each *eut* locus of *K*. *pneumoniae* towards EA metabolism under aerobic and anaerobic conditions as well as the regulatory elements which dictate the expression of each *K*. *pneumoniae eut* locus. Next, we characterized the contribution of each locus to GI colonization using a murine model with intact microbiota. Finally, we determined the distribution and conservation of these loci across the *K*. *pneumoniae* Species Complex (*KpSC*) [32].

## Results

### Ethanolamine loci of *K*. *pneumoniae* provide distinct EA-dependent growth dynamics

To identify the importance of each *eut* locus in nutrient utilization, we constructed an in-frame deletion mutant of the *eutC* gene in the long locus (*ΔeutC*_*L*_; intact short locus), the short locus (*ΔeutC*_*S*_; intact long locus), and in both loci (*ΔeutC*_*L*_*/C*_*S*_), effectively inhibiting the ability of *K*. *pneumoniae* to synthesize the ethanolamine ammonia-lyase. Despite both EutC_L_ and EutC_S_ having only 51% similarity with each other, their predicted structures were tightly aligned with an RMSD of 1.004 Å and both predicted to metabolize EA into ammonia and acetaldehyde [33,34].

Wild-type (WT) KPPR1S (ST493) [35] and each isogenic mutant strain were grown aerobically in control M9 Minimal Media (MM) supplemented with glycerol and ammonia as the sole carbon and nitrogen source, respectively. The mutant strains grew as WT in the absence of EA (**S1A Fig)**. Subsequently, when EA was furnished as the sole nitrogen source in M9 minimal media (MM), *ΔeutC*_*S*_ exhibited delayed growth during the exponential phase. In contrast, *ΔeutC*_*L*_ behaved as WT (**Fig 1Ci**), suggesting that the short locus is important for exponential growth when EA is provided as the sole nitrogen source, under aerobic conditions. Conversely, *ΔeutC*_*L*_ was unable to grow when EA was provided as the sole carbon source, while *ΔeutC*_*S*_ growth was unimpeded **(Fig 1Ciii)**. This result signifies the importance of the long locus for aerobic growth when EA is provided as a carbon source. Robust growth was observed when EA was furnished at 20 mM as a carbon source compared to 2.5 mM EA (**S1B Fig**). The WT strain grew when EA was provided as the sole carbon and nitrogen source, with the *ΔeutC*_*S*_ mutant behaving as WT (**S1C Fig**). Thus, the EA loci of *K*. *pneumoniae* provide distinct growth advantages during *in vitro* aerobic conditions depending on the context of EA as an energy source.

To provide further insight into the contribution of each locus in EA metabolism, we performed co-culture experiments under aerobic growth conditions. Mirroring the results shown in **S1A Fig**, no out-competition was observed between the WT and the mutant strains when tested under control growth media (M9 glycerol [Gly] + ammonia) (**S2A Fig**), providing further evidence that there is no intrinsic growth defect within the mutant strains. When EA was supplied as the sole nitrogen source, the WT strain outcompeted the *ΔeutC*_*S*_ and *ΔeutC*_*L*_ mutants. All strains grown with EA as a nutrient source included 200nM B12. However, the *ΔeutC*_*S*_ mutant was outcompeted earlier and to a greater extent than the *ΔeutC*_*L*_ mutant by the WT (**S2B Fig**). Thus, unlike the single strain growth kinetic studies (**Fig 1C**), our competitive growth studies reveal that both loci are critical for optimal growth when EA is provided as a nitrogen source, albeit the short locus providing a bigger advantage with EA as a nitrogen source. Corresponding to the single strain growth kinetics in **Fig 1Ciii**, when EA was provided as the sole carbon source, the WT strain was unable to rescue the complete growth defect of the *ΔeutC*_*L*_ mutant, implying the importance of the long locus in EA utilization as a carbon source (**S2D Fig**). We did not observe any difference between the WT and *ΔeutC*_*S*_ when EA was the sole carbon and nitrogen source (**S2C Fig**). Additionally, two other genetically distinct *K*. *pneumoniae* isolates, hvKP1 (ST86), a hypervirulent strain, and AZ99 (ST1322), were able to metabolize EA as a nutrient source albeit with different kinetics which resulted in variance in growth (**S1D and S1E Fig**), suggesting that the ability to utilize EA is likely a common trait across *K*. *pneumoniae* isolates.

Co-culture growth experiments between the *ΔeutC*_*L*_ mutant and the *ΔeutC*_*S*_ mutant with EA as either the nitrogen or carbon source further supported the distinct importance of each locus. With EA as the sole nitrogen source, *ΔeutC*_*L*_ (intact *eut* short locus) outcompeted *ΔeutC*_*S*_ (intact *eut* long locus), whereas *ΔeutC*_*S*_ outcompeted *ΔeutC*_*L*_ when EA was provided as the sole carbon source **(S2E Fig)**. Our growth kinetics data suggest that both *eut* loci are functional and provide a context-dependent growth advantage, with the short locus primarily used for metabolism of EA as a nitrogen source.

Oxygen availability affects the metabolic activity of facultative anaerobic bacteria [42]. As EA is present in the hypoxic environment of the gut (< 10 mmHg in the luminal colon) [36], we hypothesized that *K*. *pneumoniae* would nevertheless be able to metabolize EA as a nutrient source under anaerobic conditions. Thus, we performed anaerobic *in vitro* growth kinetic studies with EA provided as different energy sources. Single and double *eut* locus mutants grew as WT in control M9 MM (**S1F Fig**), albeit at a lower final cell density when compared to aerobic conditions **(S1A Fig)**. When EA was provided as the sole nitrogen source, both *ΔeutC*_*S*_ and *ΔeutC*_*L*_ exhibited delayed growth relative to WT. The *ΔeutC*_*S*_ mutant had a more extended lag phase than *ΔeutC*_*L*_ and the WT strain but eventually reached the same final cell density as the other two strains (**Fig 1Cii**). Our data suggest that both *eut* loci are required for optimum growth under anaerobic conditions with EA as a nitrogen source. Surprisingly, neither the WT nor the *ΔeutC*_*S*_ mutant strain could grow when EA was provided as the sole carbon source (**Fig 1Civ**). Our aerobic and anaerobic growth studies in M9 MM demonstrate that *K*. *pneumoniae* can utilize EA, with both loci contributing toward EA metabolism, without incurring a biological cost. Additionally, the ability of *K*. *pneumoniae* to metabolize EA as a carbon source depends on oxygen availability.

### A subset of gut commensals can metabolize EA

**Fig 2:**
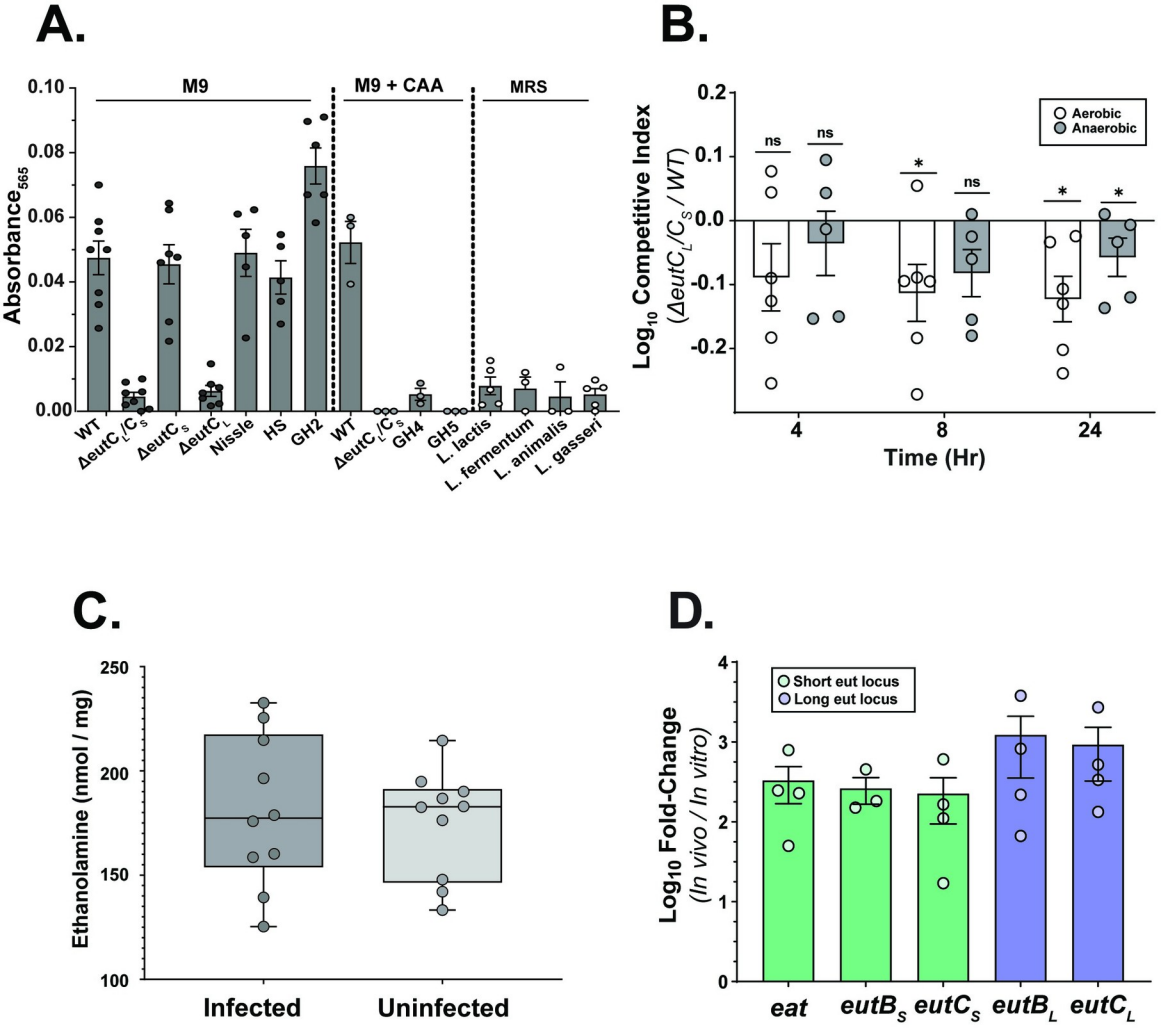
EA present in the gut is metabolized by gut commensals, and it provides *K*. *pneumoniae* with a growth advantage. **(A)** EA metabolizing capacity of *K*. *pneumoniae* WT, the isogenic mutants *ΔeutC*_*L*_*/C*_*S*_, *ΔeutC*_*S*_, *ΔeutC*_*L*_, and gut commensals using an agar plate colorimetric assay detecting acetaldehyde, a byproduct of EA metabolism. Strains were grown for 20 hours at 37°C on M9 MM (0.4% Gly, 10 mM NH_4_Cl), M9 MM with casamino acids (CAA), and De Man–Rogosa–Sharpe (MRS) agar, all containing 10 mM EA, and acetaldehyde quantified as described in *Material and Methods* section. *E*. *coli* Nissle (Nissle), *E*. *coli* healthy scientist (HS), murine *E coli* isolate (GH2), *Acinetobacter sp*. (GH4), *Acinetobacter junii* (GH5), *Lactococcus lactis* (*L*. *lactis*), *Lactobacillus fermentum* (*L*. *fermentum*), *Lactobacillus animalis* (*L*. *animalis*), and *Lactobacillus gasseri* (*L*. *gasseri*). Mean ± SEM for ≥ 3 independent experiments shown. (**B**) *In vitro* competition experiments between the WT and the *ΔeutC*_*L*_*/C*_*S*_ *K*. *pneumoniae*. Both strains were inoculated 1:1 in cecal filtrate (CF), and grown at 37°C under aerobic or anaerobic conditions. CFU were enumerated, and CI values determined at 4, 8 and 24 hours post-inoculation. Shown is mean ± SEM from ≥ 3 independent experiments. Statistical significance of CIs were calculated using Wilcoxon signed-rank test with a theoretical median of 0. **(C)** LC MS/MS data showing the concentration of EA in the colonic extracts of mice either mock-infected (uninfected) or infected with *K*. *pneumoniae*. Boxes and whiskers with data points from individual mice shown with mean and minimum to maximum values. Each symbol represents a single mouse. **(D)** qRT-PCR showing *eut* loci expression in the murine GI tract. Shown is fold-change in transcription of *eat*, *eutB*_*S*_, *eutC*_*S*_ (short *eut* locus) and *eutB*_*L*_, *eutC*_*L*_ (long *eut* locus) comparing RNA isolated from cecal contents from either *K*. *pneumoniae*-inoculated mice or grown in M9 MM. Mice were inoculated by oral feeding of 10^6^ CFU, and cecal contents harvested 15 days post-inoculation. KPPR1S 16S (*rrsA*)-specific primers were used as housekeeping gene for *2*^*-ΔΔC*^_*T*_ analysis. The values were further normalized to *gyrA* expression. For each biological replicate (n ≥ 3 for *in vitro* and *in vivo* samples), qRT-PCR was conducted in triplicate. Shown is mean ± SEM. *, *P ≤ 0*.*05*; ns, not significant.

The ability to efficiently metabolize EA was initially believed to be a trait of pathogens [37]. However, recent findings have demonstrated that commensal isolates can also metabolize EA [22,38]. Accordingly, we compared EA metabolism between *K*. *pneumoniae*, human commensal strains and those isolated from the murine GI tract by measuring acetaldehyde, a byproduct of EA metabolism (**Fig 1A)**. Our plate-based aerobic metabolism assay demonstrated that the *K*. *pneumoniae* WT strain can metabolize EA at a similar rate to the commensal *E*. *coli* isolates Nissle, HS, and a murine GI isolate, GH2 (**Fig 2A**). *The eut* double locus mutant (*ΔeutC*_*L*_*/C*_*S*_) produced negligible acetaldehyde levels, confirming that the majority of acetaldehyde is generated through EA metabolism. A comparison of acetaldehyde produced by the single *eut* locus mutants implicated the long locus as primarily responsible for acetaldehyde’s production. *Acinetobacter* strains GH4 and GH5 were either unable to produce or produced low levels of acetaldehyde. Similarly, gram-positive species isolated from the murine gut produced exceedingly low levels of acetaldehyde (**Fig 2A**).

Our previous study showed that homogenized cecal filtrate (CF) can be a gut mimetic media for competitive growth studies [16]. Liquid chromatography with tandem mass spectrometry (LC-MS/MS) analysis of CF revealed that it contained ~3.5 ng/μl of ethanolamine, indicating the retention of small metabolites that promote growth. In contrast to growth studies with M9 MM, where no out-competition was observed between the WT and the *ΔeutC*_*L*_*/C*_*S*_ mutant (**S2A Fig**), the WT consistently outcompeted the *ΔeutC*_*L*_*/C*_*S*_ mutant under both aerobic and anaerobic growth conditions in CF (**Fig 2B**). Our results provide further evidence that certain commensal isolates can metabolize EA which confers a competitive advantage in CF.

### The *eut* loci of *K*. *pneumoniae* are upregulated in the GI tract

Enteric pathogens metabolize EA present in the GI tract [21] and have been shown to modulate the gut EA level [27]. As CF provided the WT with a competitive advantage against the EA metabolism mutant (*ΔeutC*_*L*_*/C*_*S*_), we sought to determine whether *K*. *pneumoniae* gut colonization impacts the level of EA in the GI tract. We employed our murine model of *K*. *pneumoniae* gut colonization and used LC-MS/MS analysis to compare the abundance of EA in colonic samples between mock (uninfected) and WT *K*. *pneumoniae*-infected mice at 14 days post-infection. A difference in levels of EA between infected and uninfected mice was not observed **(Fig 2C)**, suggesting that, unlike *Salmonella enterica serovar Typhimurium* (*S*. *Typhimurium*), *K*. *pneumoniae* does not reduce the pool of free EA in the GI tract [27].

As our results indicate the presence of EA in the murine gut and our *in vitro* growth assays show that *K*. *pneumoniae* can metabolize EA (**Fig 1C**), we next ascertained whether *K*. *pneumoniae eut* loci respond to EA present in the GI tract, using quantitative reverse-transcription PCR (qRT-PCR) on RNA isolated from cecal samples from mice infected with WT *K*. *pneumoniae*. *eut* expression was measured by using primers that specifically targeted *eutB* and *eutC* for each locus (the two components of the ethanolamine ammonia-lyase) and *eat* (the EA importer of the short locus). We observed upregulation of transcripts from each locus, suggesting both *eut* loci within *K*. *pneumoniae* are activated (**Fig 2D**), likely due to the presence of EA in the gut. Thus, our data show that compared to *in vitro* conditions *K*. *pneumoniae eut* loci transcripts increase in the gut, though levels of EA are not affected by *K*. *pneumoniae*.

### The *eut* loci of *K*. *pneumoniae* are required for robust GI colonization

**Fig 3:**
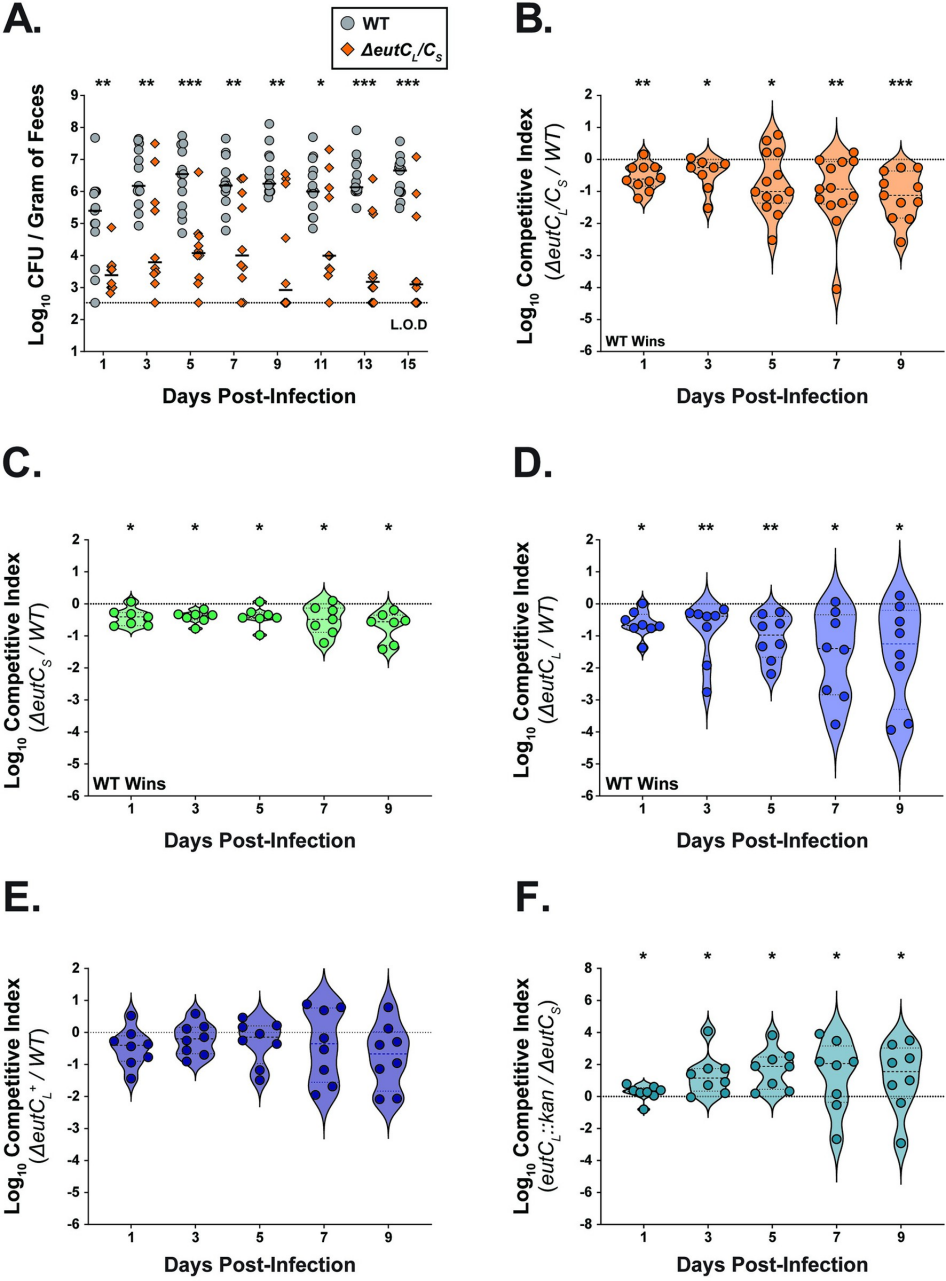
EA metabolism provides *K*. *pneumoniae* an advantage in the GI tract. **(A)** Fecal shedding of *K*. *pneumoniae* inoculated mice. Mice were orally inoculated with 10^6^ CFU of the WT *K*. *pneumoniae*, or the isogenic double locus mutant (*ΔeutC*_*L*_*/C*_*S*_) (n ≥ 10 for each group). On the indicated days, *K*. *pneumoniae* was enumerated from collected feces. Each symbol represents a single mouse on a given day. Bars, indicate medial bacterial shedding, and dashed line indicates limit of detection. A Mann-Whitney *U* test was performed between the WT and the *ΔeutC*_*L*_*/C*_*S*_ at each time point. **(B-F)** Fecal shedding from *in vivo* competition experiments. Mice were orally inoculated with a 1:1 mixture of the WT and the *ΔeutC*_*L*_*/C*_*S*_ strain **(B)**, the WT and the *eut* short locus mutant *ΔeutC*_*S*_ strain **(C)**, the WT and the *eut* long locus mutant *ΔeutC*_*L*_ strain **(D)**, the WT and the chromosomally complemented strain (*eutC*_*L*_^*+*^) **(E)**, the long locus mutant (*ΔeutC*_*L*_::*kan*) and the short locus mutant (*ΔeutC*_*S*_) **(F)**, and fecal shedding was enumerated on the indicated days (n ≥ 8). The competitive index (CI) for each day was determined as described in *Materials and Methods* section. Each symbol represents a single mouse on a given day. Values above the dashed line indicate the mutant outcompeted the WT, whereas values below the dashed line indicate WT outcompeted the mutant. Bars indicate median value. Statistical significances of CIs were calculated using Wilcoxon signed-rank test with a theoretical median of 0. *, *P ≤ 0*.*05*; **, *P ≤ 0*.*01*; ***, *P ≤ 0*.*001*.

To evaluate the significance of EA metabolism on GI colonization, we orally inoculated mice with intact microbiota with either the WT or our *ΔeutC*_*L*_*/C*_*S*_ isogenic mutant strain. We measured bacterial burden through fecal collection for 15 days as a non-invasive metric of GI colonization. Organ colonization was measured at the experimental endpoint. The *ΔeutC*_*L*_*/C*_*S*_ mutant consistently shed at a lower level compared to the WT throughout the study **(Fig 3A)** and had lower colonization levels in the oropharynx and the lower intestinal sites at the terminal time point **(S3A and S3B Fig)**. These results suggest that EA metabolism contributes to *K*. *pneumoniae* gut colonization. Next, as our murine model represents *K*. *pneumoniae* gut colonization in the presence of an intact microbiome [39], we determined whether the *ΔeutC*_*L*_*/C*_*S*_ mutant colonization defect was microbiota-dependent. Mice were treated with ampicillin one day prior to inoculation with either the WT or *ΔeutC*_*L*_*/C*_*S*_, which effectively ablated the microbiota, allowing the experimental strains to persist due to *K*. *pneumoniae’s* intrinsic resistance to ampicillin and reach a "supershedder" state. The supershedder state entails the expansion of *K*. *pneumoniae* to high levels in the gut, allowing it to shed over 100-fold more than mock-treated mice [40]. No statistical difference in shedding was observed between the WT and the *ΔeutC*_*L*_*/C*_*S*_ mutant, with both strains shedding at a supershedder state **(S3C Fig)**, suggesting that EA metabolism is dispensable with a reduced gut microbiome afforded by antibiotic treatment that likely allows *K*. *pneumoniae* access to more preferred energy sources. Therefore, our shedding data demonstrates that the difference in colonization observed between the WT and the *ΔeutC*_*L*_*/C*_*S*_ mutant is due to an intact resident microbiome, which likely exerts enhanced nutritional competition manifesting as CR. Furthermore, we conducted competition studies to investigate the contribution of each *eut* locus to gut colonization by *K*. *pneumoniae*. Our other goal was to determine whether the parental WT could rescue the colonization defect of the *ΔeutC*_*L*_*/C*_*S*_ mutant strain. In these studies, the mice were inoculated with a 1:1 mixture of the WT strain and either the *ΔeutC*_*L*_*/C*_*S*_, the *ΔeutC*_*L*_, or the *ΔeutC*_*S*_ mutant. Shedding post-inoculation was monitored daily for up to 10 days, and competitive index (CI) was calculated. *ΔeutC*_*L*_*/C*_*S*_ was outcompeted by the parental strain, suggesting that the WT is unable to rescue the colonization defect of the EA metabolism mutant **(Fig 3B)**. Our data indicate that both *eut* loci contribute to *K*. *pneumoniae* gut colonization. The long locus mutant (*ΔeutC*_*L*_) appeared to be outcompeted more effectively than the short locus mutant (*ΔeutC*_*S*_) **(Fig 3C and 3D)**. Subsequently, chromosomal complementation at the native locus of the *ΔeutC*_*L*_ mutant competed efficiently against the WT strain in the murine GI tract, suggesting that the defect in gut colonization was specific to the disruption in EA metabolism **(Fig 3E)**.

Based on our *in vivo* CI results, we postulated that the long locus is more beneficial in aiding *K*. *pneumoniae* to overcome CR than the short locus. However, when mice were co-infected with a 1:1 mixture of *ΔeutC*_*S*_ and *ΔeutC*_*L*_ mutant strains, the *ΔeutC*_*L*_ mutant (intact short *eut* locus) outcompeted *ΔeutC*_*S*_ (intact long *eut* locus) **(Fig 3F)**, highlighting the importance of the short *eut* locus. Collectively, these data suggest that the ability to metabolize EA is critical for robust gut colonization. While both loci contribute to nutrient competition against commensals in the GI tract, the long locus considerably impacts competition when the WT strain is present.

### Unique regulators control each *eut* locus within *K*. *pneumoniae*

**Fig 4:**
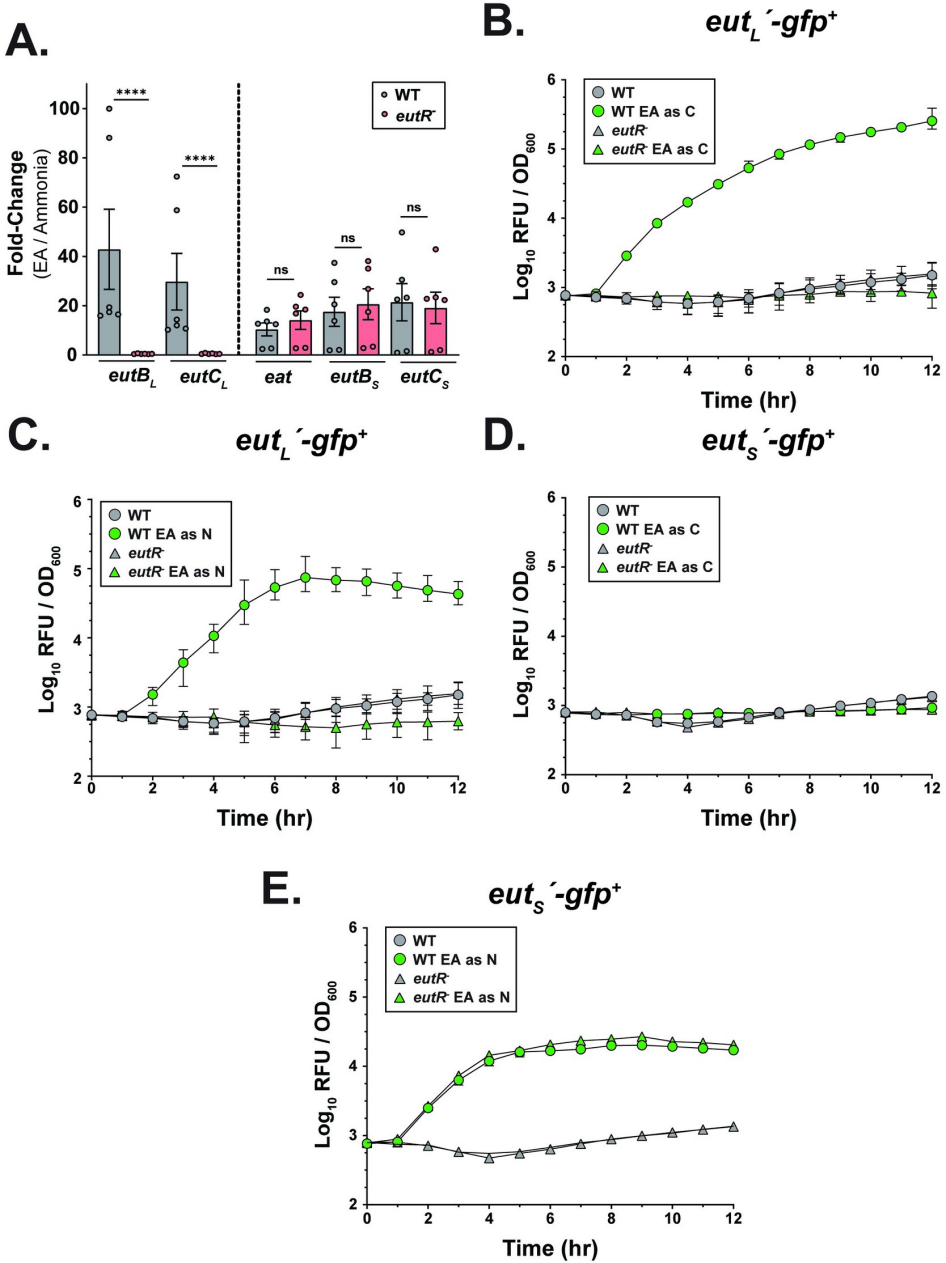
***K*. *pneumoniae* EutR regulates the long but not the short *eut* locus (A)** *In vitro* qRT-PCR measuring the transcripts of genes from the *eut* long (*eutB*_*L*_ & *eutC*_*L*_) and short (*eat*, *eutB*_*S*_, *eutC*_*S*_) locus present in the WT and the *eutR*^*-*^ isogenic mutant. Strains were either grown with EA provided as a nitrogen source (test) or ammonia (control). KPPR1S *gyrA*-specific primers were used as housekeeping gene for *2*^*-ΔΔC*^_*T*_ analysis. Mann-Whitney *U* test was performed comparing *eut* loci transcript abundance in the WT and *eutR*^*-*^ strain background. **(B-C)** GFP kinetic assay of the WT and the *eutR*^*-*^ mutant strain carrying plasmid with *eut*_*L*_ promoter transcriptional *gfp* fusion (*eut*_*L*_*΄-gfp*^*+*^). **(D-E)** GFP kinetic assay of the WT and the *eutR*^*-*^ mutant strain carrying plasmid with *eut*_*S*_ promoter transcriptional *gfp* fusion (*eut*_*S*_*΄-gfp*^*+*^). **(B&D)** Strains were grown in M9 MM + 0.4% Gly and 10 mM NH_4_Cl or M9 MM + 20 mM EA and 10 mM NH_4_Cl (EA as C). **(C&E)** Strains were grown in M9 MM + 0.4% Gly and 10 mM NH_4_Cl or M9 MM + 0.4% Gly and 2.5 mM EA. All strains grown with EA as a nutrient source included 200nM B12. ****, *P ≤ 0*.*0001*; ns, not significant.

EutR regulates the expression of the long *eut* locus in other enteric pathogens [29,41], but its contribution to *K*. *pneumoniae eut* loci regulation is unclear. We identified a putative EutR binding site in the promoter region of the long *eut* locus. To ascertain whether EutR regulates *K*. *pneumoniae eut* loci, we performed qRT-PCR on samples grown in M9 MM with EA as the sole nitrogen source. Additionally, we constructed plasmids where the promoter region of the short and the long *eut* locus was cloned upstream of green fluorescent protein (*gfp*) allowing us to perform kinetic GFP expression studies. When EA was provided as a nitrogen source, both *eut* loci were upregulated **(Fig 4A)**. Our qRT-PCR and GFP assays suggest that in a *eutR*::*cam* (*eutR*^-^) mutant background, the long *eut* locus expression was abrogated (**Fig 4A–4C**), whereas no significant change in expression of the *eut* short locus was observed (**Fig 4A, 4D and 4E**). The short locus expression was only upregulated when EA was provided as a nitrogen source, consistent with our kinetic growth and competition studies (**Figs 1C and S2E**), suggesting that the short locus facilitates EA metabolism as a nitrogen source.

**Fig 5:**
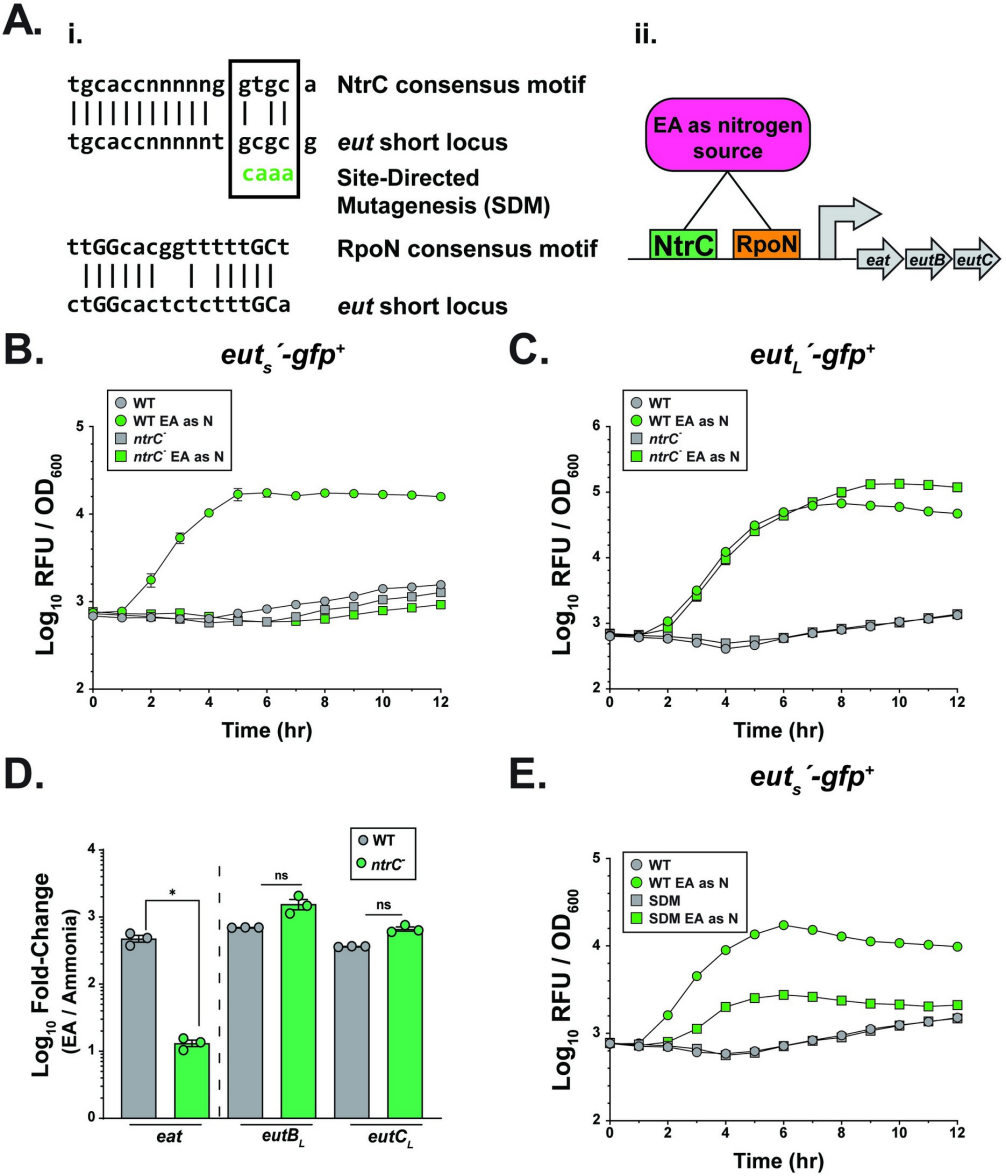
*K*. *pneumoniae eut* short locus expression is regulated in an NtrC-dependent manner. **(A)** (**i**) Shown are the putative NtrC and RpoN binding sites present in the promoter region upstream of the *eut* short locus. The conserved NtrC and RpoN binding elements are shown, with the conserved -12(GC) and the -24 (GG) RpoN binding sites capitalized. NtrC binding site selected for site-directed mutagenesis (SDM) highlighted within box. (**ii**) Proposed binding of NtrC and RpoN when EA is present as a nitrogen source. **(B-C)** GFP kinetic assay of the WT and the *ntrC*^-^ mutant strain carrying plasmid with gfp transcriptional fusion to either **(B)** the *eut*_*S*_ promoter (*eut*_*s*_*΄-gfp*^*+*^) or **(C)** *eut*_*L*_ promoter (*eut*_*L*_*΄-gfp*^*+*^). Strains were grown in M9 MM + 0.4% Gly and 2.5 mM EA (experimental) or M9 MM + 0.4% Gly & 10 mM NH_4_Cl (control). **(D)** qRT-PCR measuring transcript abundance of the *eut* short (*eat*) and long (*eutB*_*L*_, *eutC*_*L*_) locus in the WT and the *ntrC*^*-*^ isogenic mutant background. Strains were grown in M9 MM + 0.4% Gly and 2.5 mM EA (experimental) or M9 MM + 0.4% Gly & 10 mM NH_4_Cl (control). KPPR1S *gyrA*-specific primers were used as housekeeping gene for *2*^*-ΔΔC*^_*T*_ analysis. Mann-Whitney *U* test was performed comparing *eut* loci transcript abundance in the WT and *ntrC*^*-*^ strain background. **(E)** GFP kinetic assay of WT strain carrying plasmid with *gfp* fused to either the WT *eut*_*S*_ promoter region (*eut*_*S*_*΄-gfp*^*+*^) or the *eut*_*S*_ promoter with the putative NtrC binding element mutated through SDM. Strains were grown in M9 MM + 0.4% Gly and 2.5 mM EA (experimental) or M9 MM + 0.4% Gly & 10 mM NH_4_Cl (control). All strains grown with EA as a nutrient source included 200nM B12. Mean ± SEM for ≥ 3 independent experiments is shown. *, *P ≤ 0*.*05*; ns, not significant.

The chromosomes of *P*. *aeruginosa* and *A*. *baumannii*, unlike *K*. *pneumoniae*, only contain the *eut* short locus [25,26]. Their short locus also encodes a regulator termed EatR. However, we did not identify a regulator within the genetic region of the *eut* short locus, suggesting the possibility of alternative factors regulating the expression of the short locus. This led us to investigate the promoter region, wherein we identified putative binding sites for both NtrC and RpoN **(Fig 5Ai)**. NtrC is a transcription factor involved in activating genes associated with nitrogen stress and enhances the activity of the alternative sigma factor RpoN (σ^54^) through binding upstream of RpoN [42]. RpoN is known to promote arginine catabolism and the expression of virulence determinants [43,44]. We hypothesized that NtrC, in conjunction with RpoN, contributes to activating the short *eut* locus **(Fig 5Aii)**. To test our hypothesis, we constructed deletion mutants of each (*ntrC*::*cam* [*ntrC*^*-*^], *rpoN*: *cam* [*rpoN*^*-*^]). As the *rpoN*^-^ mutant was unable to grow in M9 MM with EA as a nitrogen source, we focused on the *ntrC*^*-*^ mutant and performed qRT-PCR and GFP kinetic studies on samples grown in M9 MM with EA as the sole nitrogen source. Both the short and long *eut* locus expression was tightly regulated, with both loci upregulated in the presence of EA as a nitrogen source (**Fig 5B and 5C**). In the *ntrC*- background, the expression of the short *eut* locus was downregulated, with the long *eut* locus expression unaffected (**Fig 5B–5D**) (**S4A and S4B Fig**). Furthermore, through site-directed mutagenesis (SDM), we replaced the conserved GCGC sequence [45] in the putative NtrC binding site at the short *eut* locus with CAAA, the least likely nucleotide sequence (**Fig 5Ai**). Although the SDM sequence did not completely abolish the GFP expression in the presence of EA, it reduced it, suggesting a direct involvement of NtrC in regulating the short *eut* locus (**Fig 5E**). Thus, our results show that the *eut* loci are upregulated in the presence of EA as a nitrogen source. In contrast, only the long *eut* locus is upregulated when EA is provided as a carbon source, suggesting context-dependent modulation of *eut* loci expression. Consistent with other *Enterobacteriaceae*, EutR of *K*. *pneumoniae* is involved in the regulation of the long *eut* locus for EA metabolism. Our data also demonstrate that when EA is metabolized as a nitrogen source, NtrC-RpoN directly modulates the expression of the *eut* short locus.

### *eut* locus conservation varies between taxa in the *K*. *pneumoniae* Species Complex

To explore if this metabolic behavior is likely conserved across the population and not specific to the isolates we tested, we surveyed the presence of both *eut* loci across the *KpSC*, which comprises *K*. *pneumoniae* and six additional closely related taxa that can each cause human disease and cannot be readily distinguished by standard clinical microbiology techniques [32]. We queried 7829 *KpSC* genomes for each *eut* gene and the binding motifs for EutR, RpoN and NtrC. The short and long *eut* loci were intact in 98.72% and 93.5% of all queried genomes, respectively. The short locus was highly conserved among all taxa, as were the RpoN and NtrC binding sites, indicating consistent regulation and a strong evolutionary pressure for maintenance of this locus. However, we observed a partial deletion of the *eat* gene in several genomes (n = 90), which was notably common among those representing ST23 (n = 79 of 93 ST23 genomes), a dominant cause of so-called ‘hypervirulent’ community-acquired *K*. *pneumoniae* infections. Further investigation revealed this was caused by a 5’ truncation of the first 323 nucleotides (108 residues), consistent with a previous report highlighting a sequence insertion within *eat* that was associated with the major globally-distributed ST23 sub-lineage, known as CG23-I [46]. It is unclear if these isolates can still use Eat as an EA permease by translating from the Met 109 as a start codon (although the first 3 transmembrane alpha-helices would be missing from the resulting protein).

**Fig 6:**
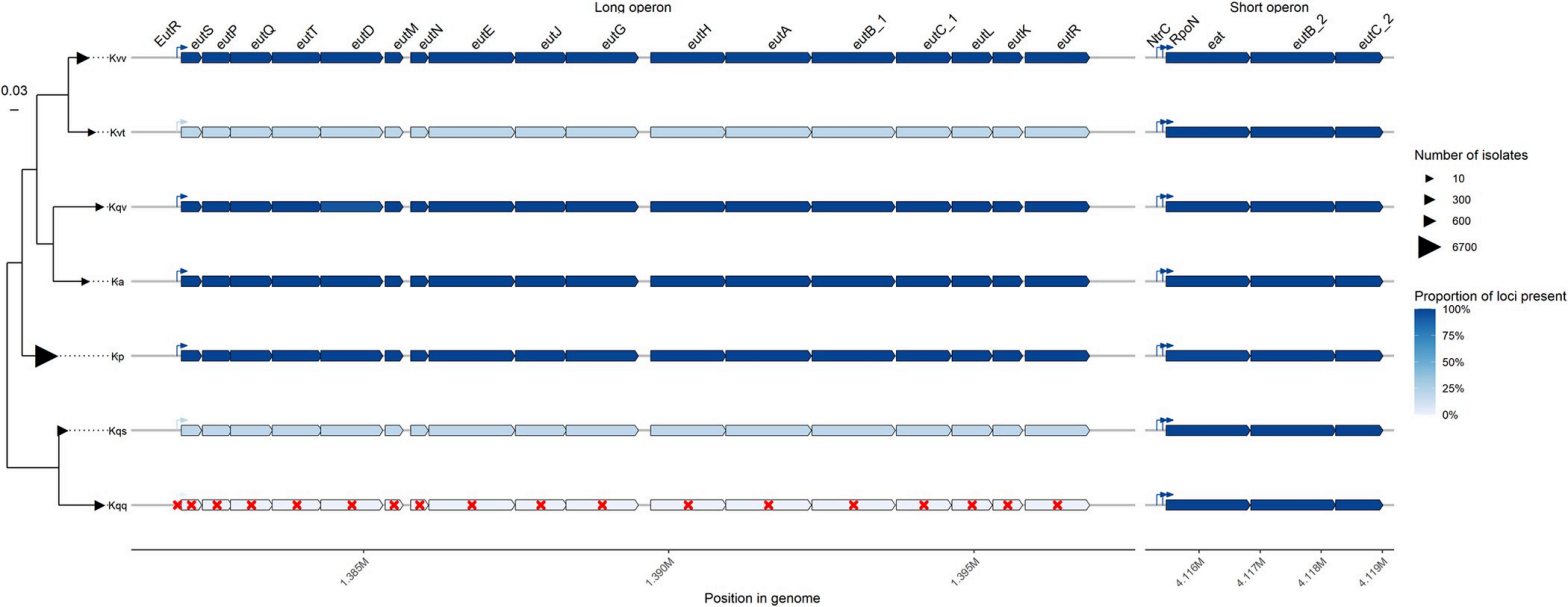
The short *eut* locus is conserved among the *K*. *pneumoniae species complex* but presence of the long locus varies by taxa. Heatmap gene arrows showing the distribution of the *eut* loci across the entire *K*. *pneumoniae* Species Complex (*KpSC*), ordered by phylogeny. The darker blue indicates higher proportion of loci presence within a given taxon. Genes are shown as arrows, while putative regulator binding sites are shown as raised directional arrows. Red crosses indicate complete absence of a genetic feature in a taxon: *Ka—K*. *africana*. *Kp–K*. *pneumoniae*. *Kqq–K*. *quasipneumoniae subsp*. *quasipneumoniae*. *Kqs–K*. *quasipneumoniae subsp*. *similipneumoniae*. *Kqv–K*. *quasivariicola*. *Kvt–K*. *variicola subsp*. *tropica*. *Kvv–K*. *variicola subsp*. *Variicola*.

**Fig 7:**
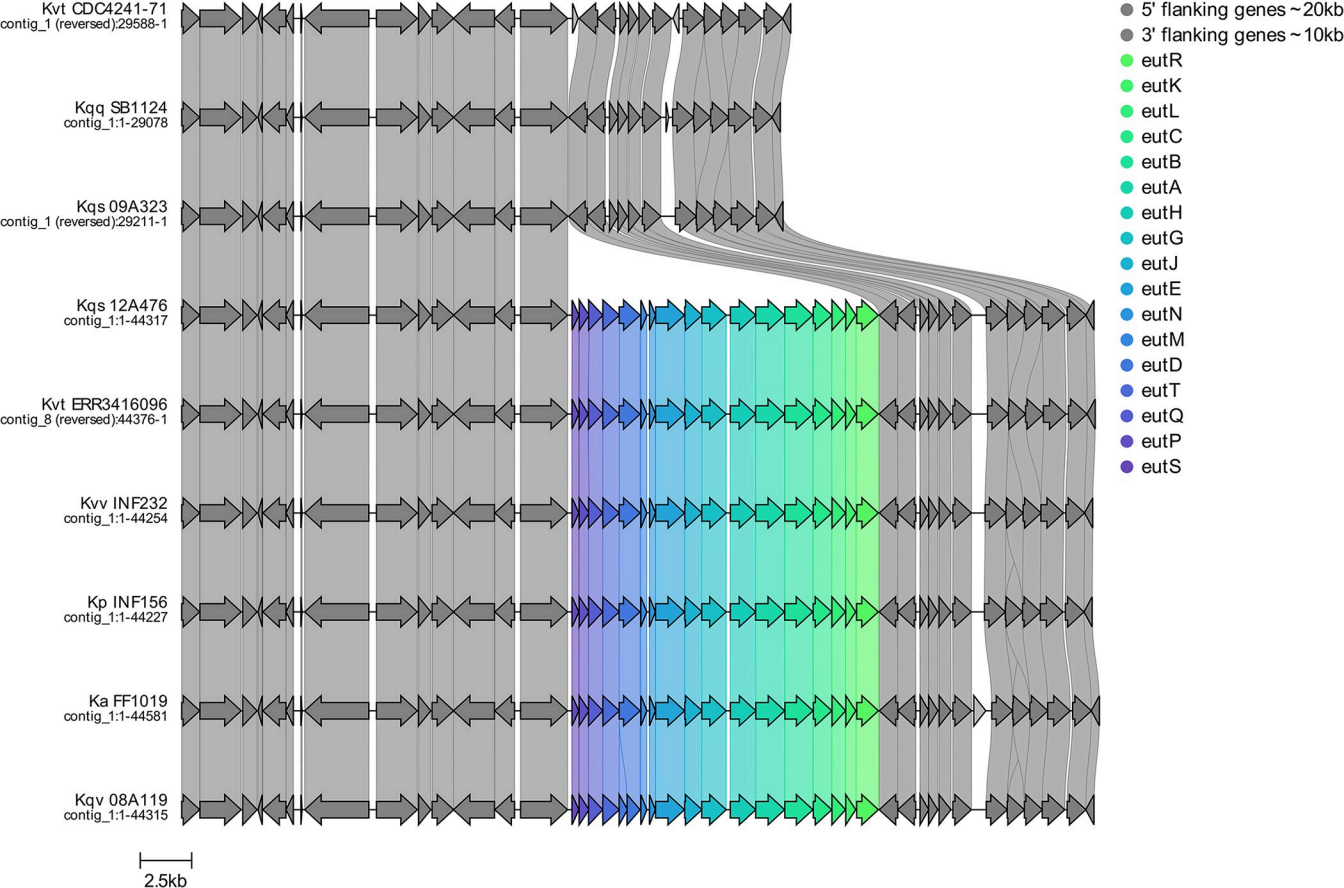
The *eut* long locus is present within a metabolism-focused region of the chromosome. Gene comparison map showing typical arrangements between the *ypfJ* and *yybH* flanking genes. Two out of 5 *K*. *variicola subsp*. *tropica* isolates lacked the operon, along with 235 out of 291 *K*. *quasipneumoniae subsp*. *similipneumoniae* genomes.19*Ka—K*. *africana*. *Kp–K*. *pneumoniae*. *Kqq–K*. *quasipneumoniae subsp*. *quasipneumoniae*. *Kqs–K*. *quasipneumoniae subsp*. *similipneumoniae*. *Kqv–K*. *quasivariicola*. *Kvt–K*. *variicola subsp*. *tropica*. *Kvv–K*. *variicola subsp*. *variicola*.

The long *eut* locus was species-specific: It was found in >99.9% *K*. *pneumoniae*, *K*. *variicola subsp*. *variicola*, *K*. *africana* and *K*. *quasivariicola* genomes; however it was present in only 19% of *K*. *quasipneumoniae subsp*. *similipneumoniae* (n = 56/291) and 60% *K*. *variicola subsp*. *tropica* (n = 3/5), and was completely absent from *K*. *quasipneumoniae subsp*. *quasipneumoniae* suggesting that it was lost from the common ancestor of the later taxon (**Figs 6 and 7**). While our dataset contains too few genomes to speculate on the *eut*_*L*_ status of the common ancestor of *K*. *variicola subsp*. *tropica*, our data suggest that the common ancestor of *K*. *quasipneumoniae* subsp. *similipneumoniae* most likely harboured *eut*_*L*,_ which has subsequently been lost multiple times (genomes harboring the long *eut* locus were distributed throughout the *K*. *quasipneumoniae* subsp. *similipneumoniae* phylogeny (**S5 Fig**) and crucially were always identified in the same genomic context, matching that of the other *KpSC* taxa (**S6 Fig**), although we cannot rule-out the possibility of a single loss in the ancestor of all *K*. *quasipneumoniae*, followed by multiple independent reacquisitions.

## Discussion

For an incoming pathobiont such as *K*. *pneumoniae* to establish itself in the GI tract, it must either outcompete the resident microbiota for energy sources and/or metabolize alternative energy sources. Here, we examined how the metabolism of EA allows *K*. *pneumoniae* to overcome CR and establish asymptomatic colonization in the GI tract. We have systematically demonstrated the ability of *K*. *pneumoniae* to utilize EA as a carbon and nitrogen source through two distinct *eut* loci. In contrast, most gut bacteria that metabolize EA contain a single locus [20,23].

As EA is considered an abundant GI nutrient source [23], we hypothesized that *K*. *pneumoniae* metabolizes EA to overcome CR and carve out its niche in the GI tract. LC-MS/MS quantified free EA within the GI tract **(Fig 2C)**, corroborating results from previous studies investigating EA utilization that showed the presence of EA in the GI tract [27,30]. Notably, *S*. *Typhimurium* induced colitis causes a reduction in free EA [27] whereas, *K*. *pneumoniae* is generally not associated with microflora disruption [39] nor observed to reduce EA levels, suggesting this effect induced by *S*. *Typhimurium* may decrease the amount of EA available in the GI tract. We identified several gut commensals that can metabolize EA, albeit to different levels. Recent studies have demonstrated that commensal gut bacteria can metabolize EA and modulate the levels of EA in the human gut, which correlates with changes in gut permeability, inflammation, and glucose metabolism [38,47].

We observed upregulation of the short and long *eut* loci within the GI tract, with *ΔeutC*_*L*_*/C*_*S*_ mutant colonizing the murine gut poorly, suggesting that the metabolism of EA expands the nutritional capacity of *K*. *pneumoniae*, allowing it to bypass nutrient limitations and establish colonization in the gut. Our co-infection studies provided insights into the contribution of each *eut* locus towards gut colonization **(Fig 3)**. When the *eut* single-locus mutants competed in a co-infection experiment, the *ΔeutC*_*L*_ and *ΔeutC*_*S*_ were outcompeted by the WT strain, implying that both loci are essential for colonization and provide a unique benefit. Furthermore, *ΔeutC*_*L*_ outcompeted *ΔeutC*_*S*_ when EA was provided as a nitrogen source *in vitro*, and in the GI tract, suggesting that *K*. *pneumoniae* preferentially metabolizes EA as a nitrogen source. This was also reflected in the presence of the two loci across the *KpSC*, where the short *eut* nitrogen utilization locus was more prevalent than the long carbon utilization locus. As most commensals that metabolize EA within the GI tract only contain the *eut* long locus [20,47], *K*. *pneumoniae* may retain the *eut* short and long locus to outcompete *eut*_*L*_*-*containing commensals. Gut commensals, particularly *Firmicutes* have an amino acid auxotrophy [48]. Thus, we postulate that *K*. *pneumoniae*, through enhanced EA metabolism, affects auxotrophic nutrient availability, leading to out-competition of commensals.

Our genomic analyses showed that the short *eut* locus is highly conserved among the *KpSC*, although the *eat* gene is truncated in the major sub-lineage of ST23 associated with hypervirulent infections that can affect otherwise healthy hosts. While the long locus was also highly conserved among *K*. *pneumoniae*, the dominant taxon identified among human infection and gut carriage isolates, it was not conserved among all taxa. Notably the long locus was absent from *K*. *quasipneumoniae* subsp. *quasipneumoniae*, which, may at least, in part explain the comparatively low frequency of its isolation from human gut carriage specimens in comparison to its sister taxon, *K*. *quasipneumoniae* subsp. *similipneumoniae* (as well as *K*. *pneumoniae* and *K*. *variicola* subsp *variicola* which together are the most frequently identified taxa).

Surprisingly, although *K*. *pneumoniae* has two *eut* loci, it can only use EA as a carbon source under aerobic conditions, signifying the importance of oxygen as a terminal electron acceptor. Besides *S*. *Typhimurium*, other enteric pathogens with a single locus cannot use EA as a carbon source under aerobic conditions [27,37]. In *S*. *Typhimurium*, acetate formed by EutD is phosphorylated by EutQ, eventually producing Acetyl-CoA, and EutQ is required for growth on EA under anaerobic conditions in the presence of respiratory electron acceptor tetrathionate (S_4_O_6_^2−^) [49]. Similar to *E*. *coli*, *K*. *pneumoniae* does not have the *ttr* locus, which encodes a tetrathionate reductase, making it unlikely that *K*. *pneumoniae* would be able to use exogenously supplied tetrathionate as an electron acceptor under anaerobic growth conditions [50]. Furthermore, *K*. *pneumoniae* colonization does not elicit an inflammatory response [39], so host inflammatory molecules are unavailable to serve as electron acceptors under anaerobic conditions. Although, *K*. *pneumoniae* gut colonization may modify the gut microbiome, increasing oxygen availability for respiration. Additionally, the host reduces inorganic dietary non-heme ferric iron (FeIII) to ferrous iron (FeII) through ferrireductases before uptake [51]. FeIII could act as an electron acceptor [52], which *K*. *pneumoniae* could use for anaerobic respiration. Further studies aiming to look at microbiome modulations that occur with *K*. *pneumoniae* and the use of different electron acceptors under anaerobic conditions would help us provide a better understanding of *K*. *pneumoniae* interactions and metabolism in the gut.

The *eut* long locus shares genetic and functional similarities, such as microcompartment formation and use of B12 as a cofactor with the propanediol utilization (*pdu*) operon [53,54]. The *pdu* operon is responsible for 1,2-propanediol catabolism, a byproduct of fucose and rhamnose fermentation [55]. We have recently shown that host mucin-derived fucose provides a growth advantage to *K*. *pneumoniae* in the murine GI tract [16]. Thus, it is likely that the fucose (*fuc*), *pdu*, and *eut* operons collectively contribute to providing a growth advantage to *K*. *pneumoniae* against the resident gut microbiota.

Asymptomatic GI colonization is an understudied aspect of *K*. *pneumoniae* that is crucial in understanding its transmission, survival, virulence, and overall pathogenicity. Herein, we demonstrate that EA derived from cell membranes is a valuable carbon and nitrogen source for *K*. *pneumoniae*, allowing it to bypass nutritional competition in the gut and overcome CR by opportunistic use of EA. In addition, we establish that each *eut* locus has distinct efficiency in metabolizing EA depending on the nutrient source context. We identify NtrC and RpoN that respond to nitrogen stress as novel regulators of the *eut* short locus, suggesting stiff competition for preferential nitrogen sources in the gut. This study advances our understanding of how an asymptomatic pathobiont overcomes CR through the metabolism of alternative energy sources, allowing us to identify potential new strategies to reduce the burden of *K*. *pneumoniae*.

## Material and Methods

### Ethics statement

This study was conducted according to the guidelines outlined by the National Science Foundation animal welfare requirements and the Public Health Service Policy on Humane Care and Use of Laboratory Animals [56]. All mouse experiments were conducted according to the guidelines of the American Association for Laboratory Animal Science (AALAS) and were approved by the Wake Forest Baptist Medical Center Institutional Animal Care and Use Committee (IACUC). The approved protocol number for this project is A20-084.

### Strain and plasmid construction

Strains, PCR primers, qRT-PCR primers, and plasmids used in this study are listed in **Tables 1, S1, and S2.** The deletion mutants (*ΔeutC*_*S*_, AZ159; *ΔeutC*_*L*_, AZ160; *ΔeutC*_*L*_*/C*_*S*_; AZ169) were constructed as previously described [39,57]. Briefly, Q5 High-Fidelity DNA Polymerase (New England BioLabs [NEB]; M0491L) was used to amplify the kanamycin (kan) cassette and the surrounding FLP recombination target sites using plasmid pKD4 as a template. The PCR product was purified and electroporated (1.8 kV, 400 Ω, 25 μF) into a KPPR1S-derivative containing the temperature-sensitive plasmid pKD46 (AZ63), with the λ red recombination genes downstream of an arabinose-inducible promoter. Recombination was performed as described [58], and successful mutants were selected on lysogeny broth (LB) agar supplemented with kan (25 μg/mL). pKD46 was removed by growing plates above the temperature sensitivity threshold (37°C), and resulting colonies (*eutC*_*S*_::*kan*, AZ157; *eutC*_*L*_::*kan*, AZ158) were confirmed via colony PCR. The kan cassettes were subsequently removed by electroporating pFlp3 that encodes a FLP recombinase and tetracycline resistance, into AZ157 and AZ158. Successful mutants (*ΔeutC*_*S*_, AZ159; *ΔeutC*_*L*_, AZ160) were selected for both tetracycline (10 μg/mL) resistance and kanamycin (25 μg/mL) sensitivity and confirmed via colony PCR. The pFlp3 was removed via growth on 5% sucrose plates. AZ169 (*ΔeutC*_*L*_*/C*_*S*_) was constructed by introducing the kan cassette into AZ160 as described above.

**Table 1:** Strains used in the study^a^.

Strain	Description	Antibiotic Resistance	Reference
AZ17	KPPR1 serotype 2, O antigen type 1, derivative of ATCC 43816	*rif* ^*r*^	[35]
AZ55	KPPR1S *str*^*r*^ derivative of KPPR1	*str*^*r*^, *rif*^*r*^	[68]
AZ63	KPPR1S pKD46	*str*^*r*^, *rif*^*r*^, *spec*^*r*^	[39]
AZ94	KPPR1; *apra*::*tn7*	*apr*^*r*^, *rif*^*r*^	[69]
AZ99	*str*^*r*^ derivative of AZ10 *K*. *pneumoniae* stool isolate, ST1322, *wzi* 372	*str* ^*r*^	[39]
AZ95	KPPR1 pKD46	*rif*^*r*^, *spec*^*r*^	This Study
AZ121	hvKP1 serotype 2 ST86 liver isolate	*amp* ^*r*^	[70]
AZ157	KPPR1S *eutC*_*S*_::*kan*	*str*^*r*^, *rif*^*r*^, *kan*^*r*^	This Study
AZ158	KPPR1S *eutC*_*L*_::*kan*	*str*^*r*^, *rif*^*r*^, *kan*^*r*^	This Study
AZ159	KPPR1S *ΔeutC*_*S*_	*str*^*r*^, *rif*^*r*^	This Study
AZ160	KPPR1S *ΔeutC*_*L*_	*str*^*r*^, *rif*^*r*^	This Study
AZ169	KPPR1S *ΔeutC*_*L*_*/C*_*S*_	*str*^*r*^, *rif*^*r*^, *kan*^*r*^	This Study
AZ195	*E*.*c* S17-1 λ pir pPROBE	*tp*^*r*^, *sm*^*r*^, *kan*^*r*^	[71]
AZ218	KPPR1 *eutC*_*L*_::*kan*	*rif*^*r*^, *str*^*r*^, *kan*^*r*^	This Study
AZ222	MKP103 *eutR*::*cam*	*cam* ^*r*^	[59]
AZ225	KPPR1 *eutR*::*cam*	*rif*^*r*^, *cam*^*r*^	This Study
AZ226	KPPR1 *rpoN*::*cam*	*rif*^*r*^, *cam*^*r*^	This Study
AZ227	KPPR1 *ntrC*::*cam*	*rif*^*r*^, *cam*^*r*^	This Study
AZ282	KPPR1S *eutC*_*L*_^*+*^	*str*^*r*^, *rif*^*r*^	This Study
AZ133	*Escherichia coli Nissle*	*-*	[72]
AZ134	*Escherichia coli HS*	*-*	[73]
AZ190	*Lactobacillus fermentum*	*-*	Acquired from R. Nagpal
AZ191	*Lactobacillus gasseri*	*-*	ATCC 33323 (DSM 20243)
AZ238	*Lactococcus lactis*	*-*	This Study
AZ239	*Lactobacillus animalis*	*-*	This Study
AZ244	*GH2 murine gut isolate E*. *coli*	*-*	This Study
AZ292	*GH4 Acinetobacter sp*.	*-*	This Study
AZ294	*GH5 Acinetobacter junii*	*-*	This Study

^a^amp^r^, ampicillin resistant; apra^r^, apramycin resistant; cam^r^, chloramphenicol resistant; kan^r^, kanamycin resistant; rif^r^, rifampicin resistant; str^r^/sm^r^, streptomycin resistant; tp^r^, trimethoprim resistant.

To construct AZ225 (*eutR*::*cam* [*eutR*^*-*^]), AZ226 (*rpoN*::*cam* [*rpoN*^*-*^]), and AZ227 (*ntrC*::*cam* [*ntrC*^*-*^]) genomic DNA was isolated from the corresponding mutant with a transposon insert in the gene of interest in the MKP103 background [59]. The transposon element with the chloramphenicol (cam) cassette was then amplified with ~500-bp homology using Q5 polymerase. λ red recombination was carried out as described above, and successful mutants in the KPPR1 background were selected on LB agar with cam (50 μg/mL) and confirmed via colony PCR.

A chromosomal complement of *ΔeutC*_*L*_ (*eutC*_*L*_^*+*^; AZ282) was constructed as described [46]. Briefly, the *eutC*_*L*_ gene with ~500bp upstream and downstream sequence was amplified from KPPR1S genomic DNA via Q5 polymerase. NotI-HF and NheI-HF were used to digest pKAS46 followed by the *eutC* PCR product cloned into pKAS46 using the NEBuilder HiFi DNA Assembly Master Mix (NEB; E2621L) and transformed into *Escherichia coli* S17-1 λpir. Successful transformants were confirmed with PCR and subsequently through Sanger sequencing. Conjugation was performed with AZ160 for *eutC*_*L*_, and successful complemented strains were selected and confirmed as described [57].

The plasmid pPROBE encoding green fluorescent protein (GFP) was used to insert the predicted promoter region of the long (*eut*_*L*_) and short *eut* (*eut*_*S*_) locus upstream of *gfp* (transcriptional fusion). The *eut*_*S*_ and *eut*_*L*_ promoter region (~500 bp) was amplified from KPPR1S genomic DNA via Q5 PCR. SalI-HF and BamHI-HF or EcoRI-HF were used to digest pPROBE and, subsequently assembled with the *eut* promoter PCR products using the NEBuilder HiFi DNA Assembly Master Mix (NEB; E2621L) and transformed into *Escherichia coli* S17-1 λpir. Successful colonies were confirmed via PCR, plasmid isolated and the insert confirmed through Sanger sequencing. Subsequently, the plasmids were transformed into *K*. *pneumoniae* strains of interest. The gcgc to caaa nucleotide substitution within the putative NtrC binding site of the *eut*_*S*_ promoter fused to *gfp* (pPROBE) was carried out using the Q5 site-directed mutagenesis kit (NEB; E0554S). Plasmid was isolated from successful transformants, and the substitution confirmed through Sanger sequencing.

### Cecal filtrate (CF) preparation

Uninfected mice were euthanized as described below with their cecum excised. A small incision was made at the distal end of the cecum and placed, cut side down, into a 2 mL screwcap tube. The contents of the cecum were squeezed into the tube and the remaining tissue was discarded. The resulting cecal contents were weighed and diluted 1:5 in PBS. 3–4 tubes were pooled into a 15 mL conical tube (SARSTEDT; 62.554.100) and pelleted at ~7,700 x *g* for 10 minutes at room temperature. The supernatant was then aliquoted into Eppendorf tubes and pelleted again at 21,000 x *g* for 10 minutes. The resulting supernatant was then transferred to a reservoir, drawn up by a 5 mL syringe (Fisherbrand; 14955458), and filter sterilized with a 0.2 μm filter (Fisherbrand; 09-719C) into 2 mL Eppendorf tubes. The resulting CF was stored at -20°C and diluted 1:2 with PBS for experimental use at a final dilution of 1:10.

### Kinetic growth assays

A single colony of each KPPR1S-derived experimental strain was added to 5 mL M9 Minimal Media (MM) with 0.4% Glycerol (Gly; sole carbon source) and 10mM NH_4_Cl (sole nitrogen source) and grown overnight at 37°C with constant agitation. The following day, cultures were washed three times in PBS, diluted 1:100 into 5 mL test media (M9 + 0.4% Gly and 10mM NH_4_Cl; M9 + 0.4% Glycerol, 2.5 mM EA, & 200nM B12; M9 + 20 mM EA, 10 mM NH_4_Cl, & 200nM B12; M9 + 25 mM EA & 200 nM B12) and grown at 37°C with constant agitation. For all studies where EA was added in the growth medium, cobalamin (B12) was also included. At designated time points, a 20 μL sample was taken, serially diluted and samples plated on selective antibiotic plates for enumeration.

For anaerobic growth kinetics, a single colony of the strain to be tested was inoculated into 5 mL M9 Gly & NH_4_Cl and grown at 37°C with constant agitation for 24 hours. Afterwards, 100 μL starter stocks were prepared in 20% glycerol and stored at -80°C. Freezer stocks were thawed, sub-cultured 1:100 in 5 mL of M9 Gly & NH_4_Cl and grown for 12 hours at 37°C with constant agitation. Next, cultures were adjusted to an OD_600_ of 3 in test media and moved into the anaerobic chamber (Coy Labs; #1200002). Each strain was diluted 1:100 in 5 mL test media and grown statically under anaerobic conditions at 37°C. Samples were removed at designated time points and plated onto selective antibiotic media plates for enumeration. The test media was moved into the anaerobic chamber to adjust the previous day.

For aerobic *in vitro* competition studies, overnight cultures were prepared as described above. Each competing strain was washed three times in PBS, diluted 1:100 into the test media, and grown at 37°C with constant agitation. At the designated time points, a 20 μL sample was removed, serially diluted, and plated on selective antibiotic plates for enumeration. The Competitive Index (CI) was calculated for each time points using the following formula where t(x) equals the time since initial subculture:

$$Log10\;CI=\frac{\left(\frac{Strain\;1\;t(x)}{Strain\;2\;t(x)}\right)}{\left(\frac{Strain\;1\;t(0)}{Strain\;2\;t(0)}\right)}$$

Competition studies in CF were performed as described [16]. Strains were grown overnight in LB, spun down, washed with PBS, and diluted 1:100 each into fresh CF. Samples were removed at designated time points, diluted and plated for enumeration. CI was calculated as described.

### Mouse inoculations for shedding, colonization, RNA isolation and mass spectrometry

All mouse experiments were conducted with C57BL/6J specific pathogen-free mice obtained from Jackson Laboratory that were bred and maintained in the animal facility within Biotech Place at Wake Forest School of Medicine. All infections were performed according to Young *et al*. [39]. Briefly, 5–7-week-old mice underwent food and water withdrawal for 4 hours prior to infection. Mice were then orally fed 50 μL of *K*. *pneumoniae* in PBS with 2% sucrose, followed by a 1-hour break before receiving another 50 μL, leading to a total infectious dose of ~10^6^ CFU. Inoculation doses were serial diluted and plated for enumeration to confirm infectious dose.

Post-infection fecal collection as a marker for GI colonization was carried out as previously described [39]. On collection days, fecal pellets were collected, weighed, diluted 1:10 in PBS (weight/volume), and homogenized with 2.7 mm glass beads using a bead mill homogenizer (Fisherbrand; 15-340-163). Samples were subsequently subjected to a brief spin in a microcentrifuge, and the fecal slurry was serial diluted and plated on selective antibiotic plates for enumeration. Shedding limit of detection: 100 CFU/mL.

At the experimental endpoint, mice were subjected to 5 minutes of CO_2_ exposure at a flow rate of 2.5 L/min followed by cardiac puncture for euthanasia. Following euthanasia, bacterial density in the oropharynx and the lower GI tract was determined via an oropharyngeal lavage and harvesting of the ileum, cecum, and colon tissue followed by homogenization. For oropharyngeal lavage the esophagus was exposed and an incision was made, allowing a gavage needle to deliver 200 μL of PBS into the esophagus and collected into an Eppendorf tube from the mouth (limit of detection: 33 CFU/mL). Sections of the ileum, cecum, and colon were harvested, weighed, and diluted 1:10 in PBS (weight/volume) prior to homogenization. Tissue homogenates were serially diluted and plated on selective plates for enumeration (limit of detection: 10^2^ CFU/mL).

For competition studies, bacterial strains were prepared with an inoculation dose to achieve a 1:1 ratio of each strain. ~1x10^6^ of each strain was delivered in the 100 μL inoculation dose. Fecal shedding and organ collection was performed as described above. The CI for fecal shedding for each day was calculated according to the formula below with the output representing the bacterial burden in CFU/g and the input representing the inoculation dose in CFU:

$$Log10\;CI=\frac{Strain(x)Output/Strain(y)Output}{Strain(x)Input/Strain(y)Input}$$

The “supershedder” phenotype was induced by administering 250 mg/L of ampicillin in drinking water 1 day prior to inoculating mice with either the WT KPPR1S strain (AZ55) or the *ΔeutC*_*L*_*/C*_*S*_ isogenic mutant (AZ169). The bacterial burden in the GI tract was determined by quantifying the CFU for each strain in fecal pellets for up to 7 days.

### Sample collection, RNA extraction, cDNA synthesis, and qRT-PCR

Overnight cultures of each experimental strain were grown in M9 + Gly & NH_4_Cl, diluted 1:100 into test media, and grown to an OD_600_ of ~0.5. Samples were then divided into 2 mL aliquots, spun down, resuspended in 500 μL 1x TE buffer (pH = 8.0), and 1 mL of RNAprotect Bacteria Reagent (Qiagen) was added. Samples were processed according to the manufacturer’s protocol and RNA extraction was performed using the TRIzol method [60]. RNA yields were measured using a NanoDrop spectrophotometer. The crude RNA was further treated with DNAse (TURBO DNA-*free* kit, Invitrogen; AM1907), precipitated overnight, and RNA eluted in DNAse-free water. cDNA was synthesized as per manufacturers protocol (BIO-RAD; #1725038). Samples were purified using the Qiagen MinElute PCR Purification Kit (Qiagen; #28004). For qRT-PCR, samples were prepared in duplicate on a CFX384 Touch real-time PCR detection system (BIO-RAD) according to iTaq Universal SYBR Green Supermix instructions with slight modifications. Reaction volume was adjusted to 11 μL with 10 ng of cDNA per well. Primers targeting *gyrA* were used as the internal control and cDNA from RNA isolated from the WT grown in M9 MM (Gly & NH_4_Cl) was used as the sample control. Fold-change in gene expression was calculated using the *2*^*ΔΔC*^_*T*_ method [61].

Bacterial RNA from murine cecum samples was isolated as previously described [16]. Briefly, the cecal contents of mice infected with KPPR1S were placed into a 2 mL screwcap tube (Fisherbrand; 02-682-558) containing glass beads, diluted 1:1 with RNAlater (Invitrogen; AM7020), homogenized, and stored at 4°C overnight. Next, equal volume chilled PBS was added, and samples were centrifuged at 700 x *g* for 1 minute at 4°C. The supernatant was transferred to a new tube, and spun down at 9,000 x *g* for 5 minutes at 4°C. The resulting pellet was resuspended in 500 μL 2x Buffer A (200 mM NaCl, 200 mM Tris base, 200 mM EDTA), 210 μL 20% SDS, and 500 μL UltraPure Phenol:Chloroform:Isoamyl Alcohol (25:24:1) (ThermoFisher; #15593031). The samples were transferred to 2 mL screwcap tubes containing 250 μL 0.1 mm silica beads (BioSpec Products) and homogenized on the bead mill followed by centrifugation at 6,800 x *g* for 3 minutes at 4°C before the aqueous layer was transferred to a new tube. An equal volume of Phenol:Chloroform:Isoamyl Alcohol was added to the samples followed by centrifugation at 18,000 x *g* for 5 minutes at 4°C. The RNA was precipitated from the samples using isopropanol. The resulting RNA was further purified, cDNA synthesized as described above. *K*. *pneumoniae eut* loci primers specificity was tested against cDNA synthesized from RNA isolated from naïve mice. Fold-change in gene expression was calculated using the *2*^*ΔΔC*^_*T*_ method [61]. KPPR1S 16S (*rrsA*)-specific primers were used as housekeeping gene for *2*^*-ΔΔC*^_*T*_ analysis. The values obtained were further normalized to *gyrA* expression.

### GFP kinetic assays

Each experimental strain was grown overnight in M9 MM (Gly & NH_4_Cl) and supplemented with 25 μg/mL kan, and 100 μL LB. Overnight cultures were OD_600_ adjusted to 4, and subsequently subcultured 1:100 in either M9 MM (Gly & NH_4_Cl) or test media. 100 μL of each sample was loaded with 8 technical replicates into a 96-well flat bottom tissue culture plate (Fisherbrand; FB012931). Afterwards, 100 μL of M9 + Gly & NH_4_Cl was added with 4 technical replicates as a control. The lid of the 96-well plate was treated with 0.2% Triton in ethanol to prevent fogging. The Synergy H1 Microplate Reader (BioTek) was used to measure the optical density OD_600_ and the fluorescence of GFP at OD_540_ (RFU) of cultures at designated time points. RFU values were normalized to OD_600_.

### Ethanolamine quantification in colonic samples and cecal filtrate

For liquid-chromatography tandem mass spectrometry (LC-MS/MS), 7–8-week-old mice, either uninfected (mock) or infected with the WT strain, were euthanized 15 days post-inoculation as described above. Roughly 1 cm colon segments containing fecal material were removed and flash-frozen in liquid nitrogen until sample preparation. Colonic contents were separated from the tissue prior to being weighed, diluted in 1 mL ultrapure water, and homogenized (Omni Beadruptor 24). Next, 100 μL 40% sulfosalicylic acid was added and after 15 minutes of incubation, samples were centrifuged and 1 mL of supernatant was combined with 300 μL 0.5 M NaHCO_3_, 200 μL 20 mg/mL dansyl chloride, and 20 μL 1 M NaOH. Samples were vortexed and left to incubate for 20 minutes in the dark. Afterwards, 20 μL of 25% NH_4_OH was added, the samples were vortexed again, and 500 μL of 5% ACN was added. Finally, the samples were dried under vacuum and resuspended in 50 μL of 5% ACN.

EA content was measured using an LCMS 8050 Triple Quadrupole Mass Spectrometer (Shimadzu) equipped with a Nexera UHPLC system. Ionization was set to the following parameters: nebulizing gas flow: 2 L/min, heating gas flow: 10 L/min, interface temperature: 300°C, DL temperature: 250°C, heat block temperature: 400°C, drying gas flow: 10 L/min. Dansylated ethanolamine MRM transitions in positive ESI mode: 294.9 > 280.1, 171.1, 157.1. EA was separated on a Zorbax Eclipse Plus C18 column (1.8 μm, 2.1 x 100 mm; Agilent, Santa Clara, CA) with a flow rate of 0.4 mL/min. Mobile phases consist of water + 0.1% formic acid and acetonitrile. The mobile phase gradient began at 5% acetonitrile and was then increased to 95% at 2.5 minutes. This was held at 95% for 1 minute before being returned to 5% and held until 4.5 minutes.

### EA metabolism screen

Test strains including the WT, *ΔeutC*_*L*_*/C*_*S*_ mutant and other commensals were grown overnight in liquid broth media. 1 mL was removed, and bacteria pelleted, and the supernatant resuspended in 1 mL PBS. 100 μl of the cell suspension was spread on to M9 MM agar plates containing 0.4% Gly, 10 mM NH_4_Cl and 10 mM EA, M9 with 0.2% casamino acids 0.4% Gly, 10 mM NH_4_Cl and 10 mM EA, or De Man–Rogosa–Sharpe (MRS) agar supplemented with 10 mM EA, and incubated for 20 hours at 37°C. Afterwards, the bacterial lawn was overlaid with 5mL of respective liquid agar containing 500 mM EA, and incubated at 37°C for 1 hour, followed by addition of 5 mL of 0.1% 2,4-dinitrophenylhydrazine in 2M HCl to the plate for 3 minutes at room temperature. The liquid was poured off and 5 mL of 5M potassium hydroxide was added to the plate and incubated at room temperature for 5 minutes. 1 mL of sample was removed and centrifuged briefly to pellet debris and 100 μl loaded in a 96-well plate in triplicate and absorbance read at 565 nm on a Synergy H1 Microplate Reader (BioTek). Commensals were grown on MRS with EA, and concurrently on MRS without EA (control). Absorbance values obtained from MRS with EA were subtracted from values obtained with MRS without EA.

### *16S r-RNA* sequencing

Molecular characterization of the bacterial isolates was carried out using the 16S rRNA gene-based PCR amplification and sequencing from the genomic DNA, as described in our previous studies [62]. Briefly, the quality and quantity of the isolated genomic DNA was measured using a NanoDrop One Spectrophotometer (Thermo Fisher), and the DNA was diluted to 20 ng/μl. The 16S rRNA gene were PCR-amplified using the universal primer pair 27F: 5’-AGAGTTTGATCMTGGCTCAG-3’ and 511R 5’-GCGGCTGCTGGCACRKAGT-3’ on a QuantStudio-3 Real-Time PCR System (Applied Biosystems). Sanger sequencing of the resulting amplicons was performed using the ABI 3730 Genetic Analyzer sequencer (Applied Biosystems). The obtained sequence data were BLAST-analyzed against the NCBI GenBank Database (www.ncbi.nlm.nih.gov/blast [ncbi.nlm.nih.gov]) for the confirmation of genus and species/strain identification of the isolates.

### Structure and genomic analysis

EutC structures were obtained from Alphafold2-predicted UnitProt entries A0A0H3GNA2 (eutC short) and A0A0H3GWG7 (eutC long) [33]. Structural alignments and RMSD values were generated using the Matchmaker function from UCSF ChimeraX (version 1.7rc202311272154) [34].

Sequence read data were obtained for diverse *KpSC* described in 29 studies (**S3 Table**), subjected to quality control using Trim Galore version 0.5.0 [63], assembled with Unicycler version 0.4.7 [64], annotated with Bakta version 1.1.1 [65] and subsampled to a representative set using PopPUNK clustering [66] combined with genome metadata as described in **S1 Data**. Species and sequence types were determined using Kleborate version 2.0.4 [32].

*eut* genes and regulator binding sites were identified using BLASTn (minimum 80% query coverage and identity, reference sequences from *K*. *pneumoniae* NTUH-K2044; accession GCF_000009885.1). To identify regulator binding sites (EutR, NtrC and RpoN), the entire upstream region of each operon was identified as above, and then the sequence was screened via a nested blastn, and filtered on 70% identity prior to manual inspection of variable sites. To analyze the genetic context of the long *eut* operon, the surrounding genomic regions were extracted from annotation files using the slice_multi_genbank.py (https://gist.github.com/bananabenana/20ff257f237d5a6e6f449fd7066577a1) and Clinker version 0.0.23 [67] was used for visualization. See **S1 Data** for full analysis details (https://doi.org/10.6084/m9.figshare.24718599.v2)

### Statistical analysis

All statistical analyses were performed using GraphPad Prism 9.4.1 (GraphPad Software, Inc., San Diego, CA) and R as described above. Comparisons between two groups were analyzed via the Mann-Whitney *U* test, comparisons between multiple groups were analyzed via the Kruskal-Wallis test with Dunn’s post-analysis, and competitive index/competition experiments were analyzed using the Wilcoxon signed-rank test.

## Supporting information

S1 Fig *In vitro* growth kinetics **(A)** of the WT, *ΔeutC*_*s*_, *ΔeutC*_*L*_, and *ΔeutC*_*L*_*/C*_*S*_ *K*. *pneumoniae* under aerobic conditions. All strains were grown in M9 MM + 0.4% Gly & NH_4_Cl. **(B)** of the WT in different concentrations of EA supplemented with B12 (200nM) under aerobic conditions. **(C)** of the WT and *ΔeutC*_*s*_ with EA as a sole carbon and nitrogen source (M9 MM + 25 mM EA). **(D-E)** Aerobic *in vitro* growth kinetics of AZ99 **(D)**, and hvKP1 **(E)** grown in control media (M9 + 0.4% Gly and 10 mM NH_4_Cl; black circle), EA as a nitrogen source (M9 + 0.4% Gly and 2.5 mM EA; grey square), EA as a carbon source (M9 + 20 mM EA and 10 mM NH_4_Cl; grey triangle) and EA as a sole carbon and nitrogen source (M9 + 25 mM EA; diamond). All strains grown with EA as a nutrient source included 200nM B12. **(F)** Anaerobic *in vitro* growth kinetics of WT, *ΔeutC*_*s*_, *ΔeutC*_*L*_, and *ΔeutC*_*L*_*/C*_*S*_. All strains were grown in M9 MM + 0.4% Gly & NH_4_Cl. N ≥ 3. Bars indicate Mean ± SEM.
(TIF)

S2 Fig *In vitro* competitive index (CI) studies. **(A)** WT *K*. *pneumoniae* and *ΔeutC*_*s*_, *ΔeutC*_*L*_, or *ΔeutC*_*L*_*/C*_*S*_ were inoculated 1:1 in M9 MM + 0.4% Gly & 10 mM NH_4_Cl. CIs were calculated at 4, 8, and 24 hours. **(B)** WT *K*. *pneumoniae* and *ΔeutC*_*s*_ or *ΔeutC*_*L*_ inoculated 1:1 in M9 MM + 0.4% Gly & 2.5 mM EA. CIs calculated at the noted time points. **(C)** WT and *ΔeutC*_*s*_ *K*. *pneumoniae* inoculated 1:1 in M9 MM + 25mM EA. **(D)** WT *K*. *pneumoniae* and *ΔeutC*_*s*_ or *ΔeutC*_*L*_ strains were inoculated 1:1 in M9 MM + 20 mM EA & 10 mM NH_4_Cl. **(E)** *In vitro* 1:1 competitive index of *ΔeutC*_*s*_ and *ΔeutC*_*L*_ *K*. *pneumoniae* grown in either M9 MM + 0.4% Gly & 2.5 mM EA (EA as N) or M9 MM + 20 mM EA & 10 mM NH_4_Cl. All strains grown with EA as a nutrient source included 200nM B12.
(TIF)

S3 Fig **(A)** Colonization density within the oropharynx of mice colonized with either WT or *ΔeutC*_*L*_*/C*_*S*_ *K*. *pneumoniae* at day 15 post inoculation. A Mann-Whitney *U* test was performed between the WT and the *ΔeutC*_*L*_*/C*_*S*_. **(B)** Colonization density within the ileum, cecum, and colon of mice colonized with either WT or *ΔeutC*_*L*_*/C*_*S*_ *K*. *pneumoniae*. CFU enumeration was performed at day 15 post inoculation. A Mann-Whitney *U* test was performed comparing the WT and the *ΔeutC*_*L*_*/C*_*S*_ at different gastrointestinal sites. **(C)** Supershedder state induced by administration of antibiotics in the drinking water (250 mg/L of ampicillin) prior to inoculation of mice with either WT or *ΔeutC*_*L*_*/C*_*S*_ *K*. *pneumoniae*. Mice were kept on the antibiotic water for the duration of the study. Dashed line represents supershedder threshold. A Mann-Whitney *U* test was performed comparing the WT and the *ΔeutC*_*L*_*/C*_*S*_ at different timepoints. *, *P ≤ 0*.*05*; **, *P ≤ 0*.*01*; ns, not significant.
(TIF)

S4 Fig GFP kinetic assay of WT and *ntrC*^*-*^ mutant strain carrying plasmid with gfp transcriptional fusion **(A)** with the *eut*_*L*_ promoter (*eut*_*L*_*΄-gfp*^*+*^) **(B)** or the *eut*_*S*_ promoter (*eut*_*S*_*΄-gfp*^*+*^). Strains were grown in either M9 MM + 0.4% Gly and 10 mM NH_4_Cl or M9 MM with 20 mM EA and 10 mM NH_4_Cl (EA as C) **(A-B)**. All strains grown with EA as a nutrient source included 200nM B12.
(TIF)

S5 Fig Maximum-likelihood phylogenetic tree of the two *K*. *quasipneumoniae* subspecies and the presence of the long *eut* operon. Tree contains additional *KpSC* species for context.
(TIF)

S6 Fig Distribution of pairwise Google Word distances within each Eut protein sequence across the *KpSC* dataset (n = 27,317,136–30,603,576 pairwise comparisons). A corresponding density plot is shown next to each boxplot to show the distribution of pairwise distances.
(TIF)

S1 Table List of primers used in the study. (DOCX)

S2 Table List of plasmids used in the study. (DOCX)

S3 Table Results of *eut* operon distributions across the *KpSC*. (XLSX)

S4 Table Data used to generate figures. (XLSX)

S1 Data Genomic analysis to identify the presence of *eut* loci and their conservation across *K*. *pneumoniae species complex*. (DOCX)
