# Supplementary figures and images for: Ethanolamine metabolism through two genetically distinct loci enables Klebsiella pneumoniae to bypass nutritional competition in the gut
Source: PLoS Pathog. 2024 May 7;20(5):e1012189. doi: 10.1371/journal.ppat.1012189 (PMC11101070; doi:10.1371/journal.ppat.1012189)

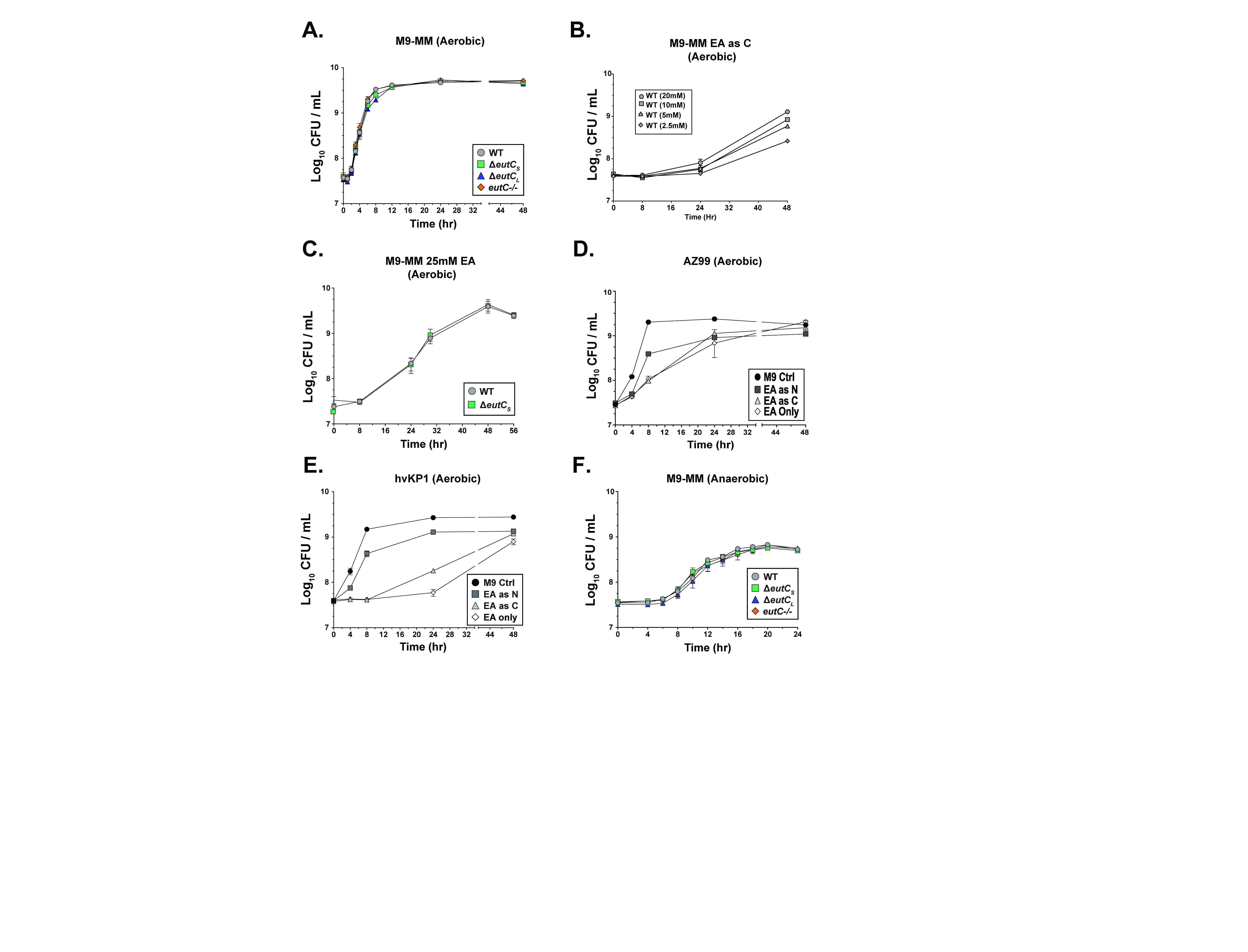

Supplement: S1 Fig — In vitro growth kinetics (A) of the WT, ΔeutCs, ΔeutCL, and ΔeutCL/CS K. pneumoniae under aerobic conditions. All strains were grown in M9 MM + 0.4% Gly & NH4Cl. (B) of the WT in different concentrations of EA supplemented with B12 (200nM) under aerobic conditions. (C) of the WT and ΔeutCs with EA as a sole carbon and nitrogen source (M9 MM + 25 mM EA). (D-E) Aerobic in vitro growth kinetics of AZ99 (D), and hvKP1 (E) grown in control media (M9 + 0.4% Gly and 10 mM NH4Cl; black circle), EA as a nitrogen source (M9 + 0.4% Gly and 2.5 mM EA; grey square), EA as a carbon source (M9 + 20 mM EA and 10 mM NH4Cl; grey triangle) and EA as a sole carbon and nitrogen source (M9 + 25 mM EA; diamond). All strains grown with EA as a nutrient source included 200nM B12. (F) Anaerobic in vitro growth kinetics of WT, ΔeutCs, ΔeutCL, and ΔeutCL/CS. All strains were grown in M9 MM + 0.4% Gly & NH4Cl. N ≥ 3. Bars indicate Mean ± SEM. (TIF) [file ppat.1012189.s001.tif]

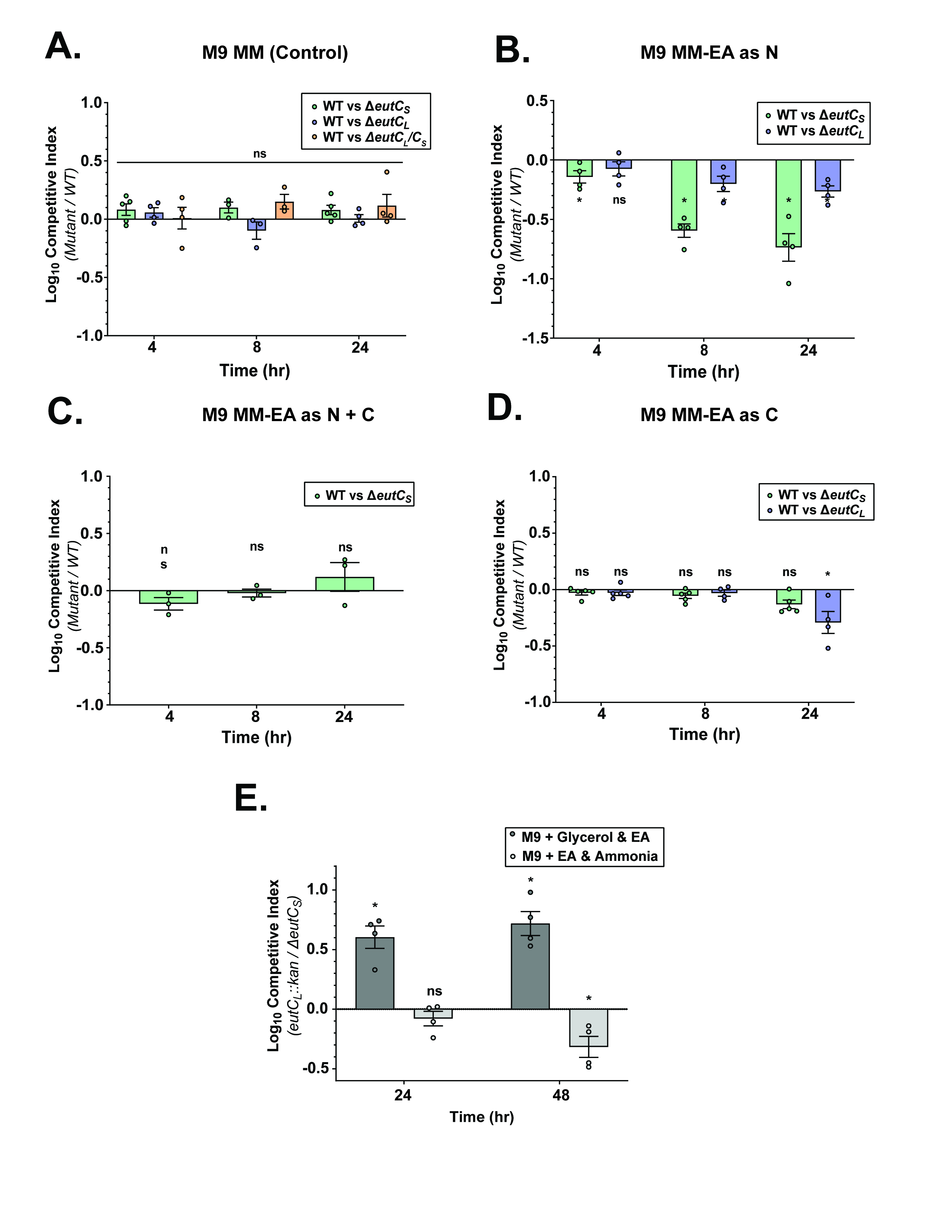

Supplement: S2 Fig — (A) WT K. pneumoniae and ΔeutCs, ΔeutCL, or ΔeutCL/CS were inoculated 1:1 in M9 MM + 0.4% Gly & 10 mM NH4Cl. CIs were calculated at 4, 8, and 24 hours. (B) WT K. pneumoniae and ΔeutCs or ΔeutCL inoculated 1:1 in M9 MM + 0.4% Gly & 2.5 mM EA. CIs calculated at the noted time points. (C) WT and ΔeutCs K. pneumoniae inoculated 1:1 in M9 MM + 25mM EA. (D) WT K. pneumoniae and ΔeutCs or ΔeutCL strains were inoculated 1:1 in M9 MM + 20 mM EA & 10 mM NH4Cl. (E) In vitro 1:1 competitive index of ΔeutCs and ΔeutCL K. pneumoniae grown in either M9 MM + 0.4% Gly & 2.5 mM EA (EA as N) or M9 MM + 20 mM EA & 10 mM NH4Cl. All strains grown with EA as a nutrient source included 200nM B12. (TIF) [file ppat.1012189.s002.tif]

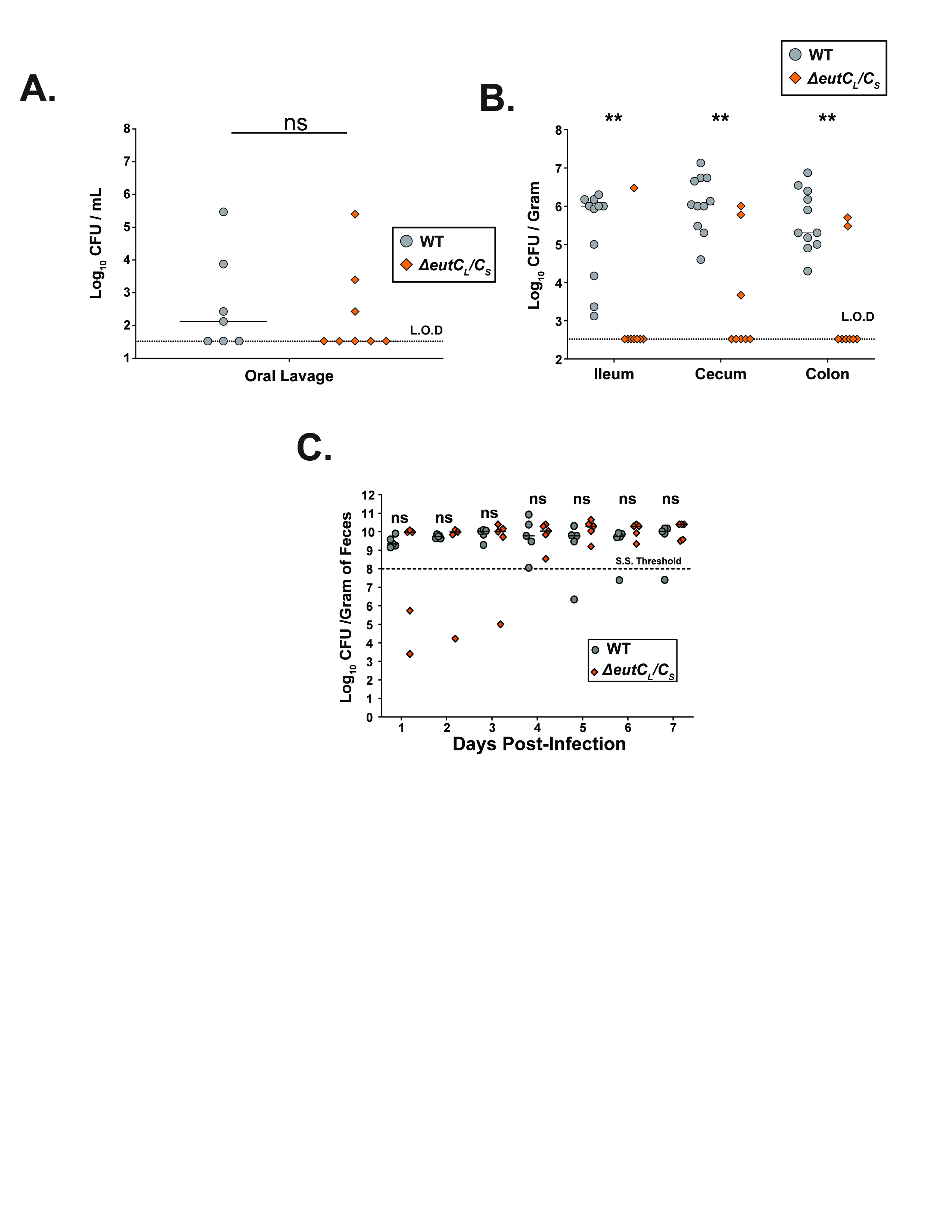

Supplement: S3 Fig — (A) Colonization density within the oropharynx of mice colonized with either WT or ΔeutCL/CS K. pneumoniae at day 15 post inoculation. A Mann-Whitney U test was performed between the WT and the ΔeutCL/CS. (B) Colonization density within the ileum, cecum, and colon of mice colonized with either WT or ΔeutCL/CS K. pneumoniae. CFU enumeration was performed at day 15 post inoculation. A Mann-Whitney U test was performed comparing the WT and the ΔeutCL/CS at different gastrointestinal sites. (C) Supershedder state induced by administration of antibiotics in the drinking water (250 mg/L of ampicillin) prior to inoculation of mice with either WT or ΔeutCL/CS K. pneumoniae. Mice were kept on the antibiotic water for the duration of the study. Dashed line represents supershedder threshold. A Mann-Whitney U test was performed comparing the WT and the ΔeutCL/CS at different timepoints. *, P ≤ 0.05; **, P ≤ 0.01; ns, not significant. (TIF) [file ppat.1012189.s003.tif]

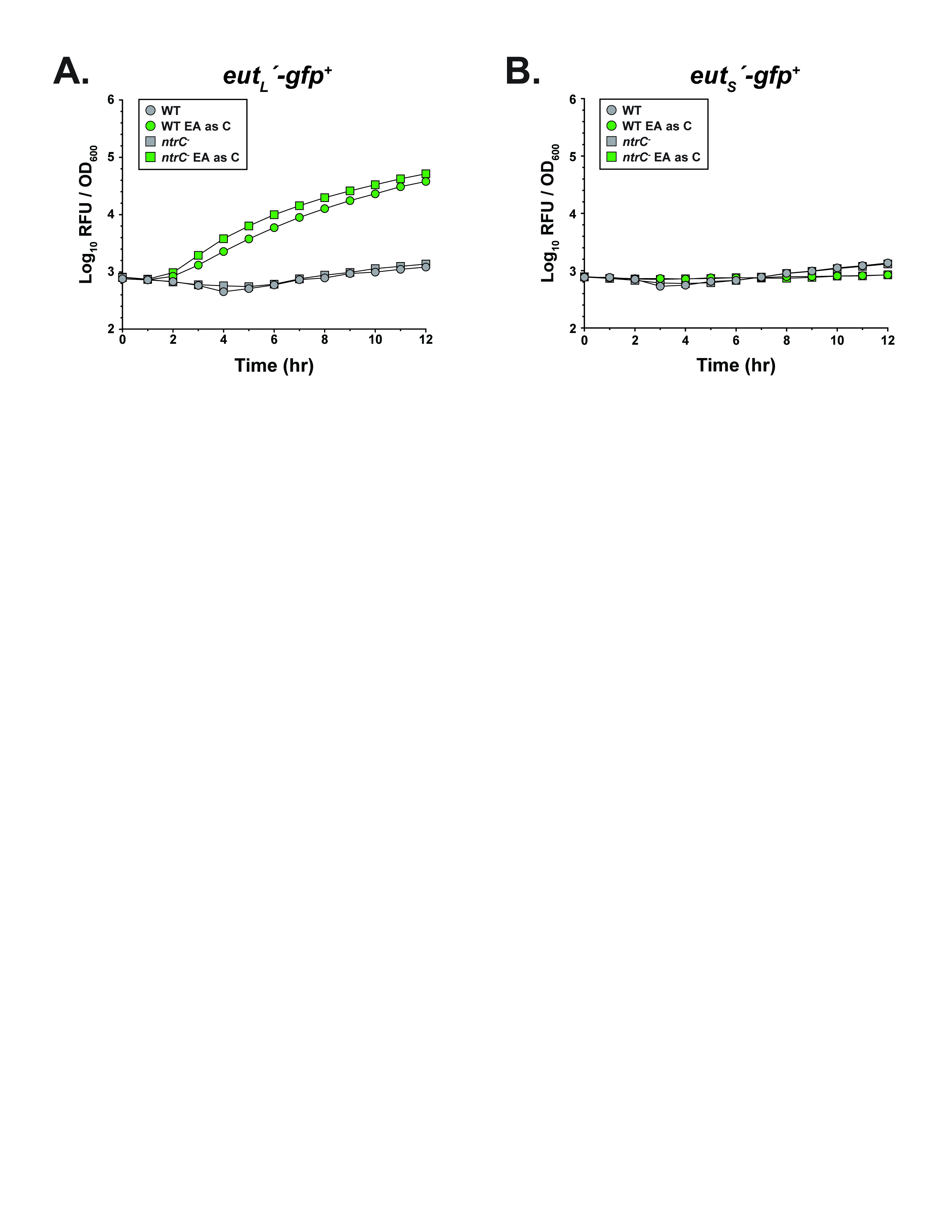

Supplement: S4 Fig — GFP kinetic assay of WT and ntrC- mutant strain carrying plasmid with gfp transcriptional fusion (A) with the eutL promoter (eutL΄-gfp+) (B) or the eutS promoter (eutS΄-gfp+). Strains were grown in either M9 MM + 0.4% Gly and 10 mM NH4Cl or M9 MM with 20 mM EA and 10 mM NH4Cl (EA as C) (A-B). All strains grown with EA as a nutrient source included 200nM B12. (TIF) [file ppat.1012189.s004.tif]

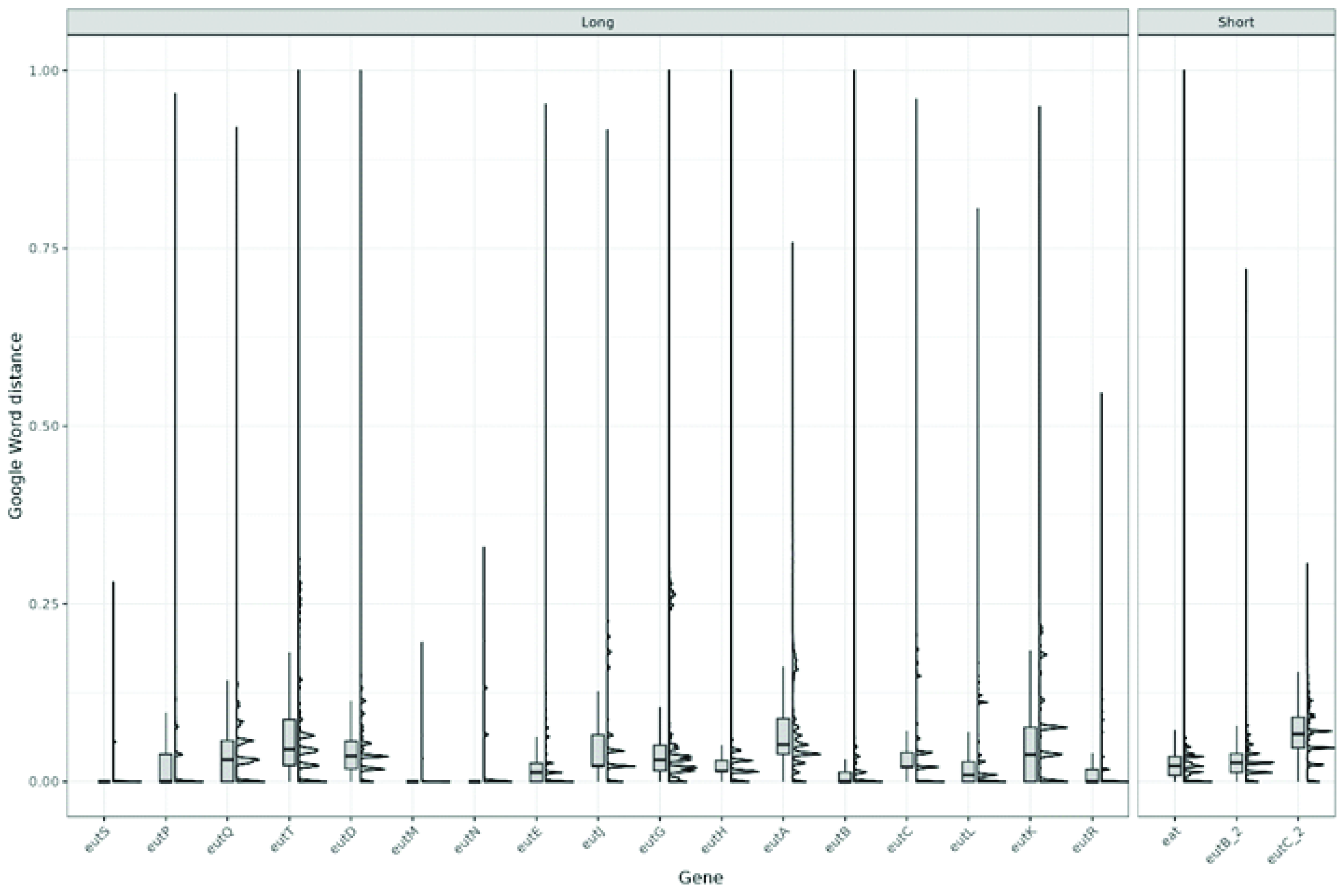

Supplement: S6 Fig — A corresponding density plot is shown next to each boxplot to show the distribution of pairwise distances. (TIF) [file ppat.1012189.s006.tif]
