# Supplementary material for: Ethanolamine metabolism through two genetically distinct loci enables Klebsiella pneumoniae to bypass nutritional competition in the gut
Source: PLoS Pathog. 2024 May 7;20(5):e1012189. doi: 10.1371/journal.ppat.1012189 (PMC11101070; doi:10.1371/journal.ppat.1012189)
Supplement: S1 Table — (DOCX) [file ppat.1012189.s007.docx]

**S1 Table.** Primers used in the study

| **Primer Name** | **Description** | **Orientation** | **Sequence** |
| --- | --- | --- | --- |
| pProbe-US | Upstream of SalI, BamH, and EcoRI restriction sites | Forward | ACGCCCGCCATAAACTGCCAG |
| pProbe-DS | Downstream of SalI, BamH, and EcoRI restriction sites | Reverse | GTTCCATGGCCAACACTTGTC |
| EutR fwd | Upstream of *eutR* gene | Forward | TGATAGTCGGTGGCGAACTG |
| EutR rev | Downstream of *eutR gene* | Reverse | ATCGGAAGCCTTACTGACGC |
| RPON fwd | Upstream of *rpoN* gene | Forward | CTTCACGACGAGGTAGAGGC |
| RPON rev | Downstream of *rpoN* gene | Reverse | ACGGCGGGATGGATAAAGAC |
| GlnG fwd | Upstream of *ntrC* gene | Forward | TTTTCCAGCTGACGGACGTT |
| GlnG rev | Downstream of *ntrC* gene | Reverse | CATCCACAAAGTCGCCGAA |
| fwd-ecoRI-SL-pr | Forward *gfp* reporter construct for *eut* short locus | Forward | ctgaattcgagctcaggccttggtacccgggatccGCATCGCCACCGACAGTTC |
| rev-SalI-SL-pr | Reverse *gfp* reporter construct for *eut* short locus | Reverse | ttagttagttagggaattaagcttctgcagtcgacACGTATTGCCTCGCCAGATAG |
| EA-Long gfp-F | Forward *gfp* reporter construct for *eut* long locus | Forward | ctgaattcgagctcaggccttggtacccgggatccGAAATGCATGGCGATGCC |
| EA-Long gfp-R | Reverse *gfp* reporter construct for *eut* long locus | Reverse | ttagttagttagggaattaagcttctgcagtcgacGACTGATTGCCCCCTGAAATC |
| Q5SDM-F | SDM in NtrC site *eut_S_* | Forward | CCCCATTTTTCAAAAGCGCCAGCGGG |
| Q5SDM-R | SDM in NtrC site *eut_S_* | Reverse | CAGGCTGCACGCTGTCGG |
| Eut-short-int-F | Upstream *eut* short locus promoter internal primer | Forward | \| CAGATGGCGCGTTTTCATGG \| \| --- \| \|  \| |
| Eut-short-int-R | Downstream *eut* short locus promoter internal primer | Reverse | CTGAACGACAGGCTGCAAAG |
| eutC-LL-pkas46-F | Upsteam primer for construction of eutC chromosomal complement | Forward | GCGCGATCGATATCAGCGCTTTAAATTTGCGCATGAATACCACCATCGGTCTGC |
| eutC-LL-pkas46-R | Downstream primer for construction of eutC chromosomal complement | Reverse | GCGCGATCGATATCAGCGCTTTAAATTTGCGCATGAATACCACCATCGGTCTGC |
| q-gyrA-F | Upstream of *gyrA* for qRT-PCR | Forward | ACACCGTCGCGTACTTTAC |
| q-gyrA-R | Downstream of *gyrA* for qRT-PCR | Reverse | GCGGGTGGTATTTACCGATTA |
| Eat-qRT-F | Upstream *eat* qRT-PCR primer | Forward | TACCTCAACGTGCAGTTTCC |
| Eat-qRT-R | Downstream *eat* qRT-PCR primer | Reverse | CCGACGCCGAGAATATTGAG |
| eutB-small-qRT-F | Upstream *eutB* short locus qRT-PCR primer | Forward | TTCTGACCCATGTGACCAATAC |
| eutB-small-qRT-R | Downstream *eutB* short locus qRT-PCR primer | Reverse | GCAGCATGGCAAGATTGATG |
| eutC-small-qRT-F | Upstream *eutC* short locus qRT-PCR primer | Forward | GATATTGGCGAAACCCTGAAAG |
| eutC-small-qRT-R | Downstream *eutC* short locus qRT-PCR primer | Reverse | GAGATACAGTTGCGCTCAGA |
| eutB-large-qRT-F | Upstream *eutB* long locus qRT-PCR primer | Forward | ATGGAAGCGCGTAACTATGG |
| eutB-large-qRT-R | Downstream *eutB* long locus qRT-PCR primer | Reverse | TGATTTGCCGGTCGTTATAGAG |
| eutC-long-qRT-F | Upstream *eutC* long locus qRT-PCR primer | Forward | AGTCCGGAAGCGATTGATG |
| eutC-long-qRT-R | Downstream *eutC* long locus qRT-PCR primer | Reverse | TTCGTAGTTGGCGGTGATG |
