# Supplementary material for: Ethanolamine metabolism through two genetically distinct loci enables Klebsiella pneumoniae to bypass nutritional competition in the gut
Source: PLoS Pathog. 2024 May 7;20(5):e1012189. doi: 10.1371/journal.ppat.1012189 (PMC11101070; doi:10.1371/journal.ppat.1012189)
Supplement: S2 Table — (DOCX) [file ppat.1012189.s008.docx]

**S2 Table.** Plasmids used in the study^a^

| **Plasmid** | **Description** | **Antibiotic Resistance** | **Reference** |
| --- | --- | --- | --- |
| pKD4 | Kanamycin cassette with FRT sites | *kan^r^* | (1) |
| pKD46 | λ red recombinase genes DS of *araBAD* promoter | *spec^r^* | (2) |
| pProbe | *Gfp* transcriptional reporter vector | *kan^r^* | (3) |
| pKAS46 | Vector for allelic exchange that contains *rpsL* for streptomycin counter-selection | *kan^r^* | (4) |
| pCre2 | Cre recombinase for the removal of loxP *cam* cassette | *amp^r^* | (5) |
| pflp3 | FLP recombinase and a tetracycline resistance and *sacB* cassette | *tet^r^* | (6) |

*^a^amp^r^, ampicillin resistant; kan^r^, kanamycin resistant; spec^r^, spectinomycin resistant; tet^r^, tetracycline resistant.*

1. Datsenko KA, Wanner BL. 2000. One-step inactivation of chromosomal genes in Escherichia coli K-12 using PCR products. Proc Natl Acad Sci U S A 97:6640-5.

2. Tyler JS, Beeri K, Reynolds JL, Alteri CJ, Skinner KG, Friedman JH, Eaton KA, Friedman DI. 2013. Prophage Induction Is Enhanced and Required for Renal Disease and Lethality in an EHEC Mouse Model. Plos Pathogens 9.

3. Miller WG, Leveau JH, Lindow SE. 2000. Improved gfp and inaZ broad-host-range promoter-probe vectors. Mol Plant Microbe Interact 13:1243-50.

4. Skorupski K, Taylor RK. 1996. Positive selection vectors for allelic exchange. Gene 169:47-52.

5. Bailey J, Manoil C. 2002. Genome-wide internal tagging of bacterial exported proteins. Nat Biotechnol 20:839-42.

6. Ramage B, Erolin R, Held K, Gasper J, Weiss E, Brittnacher M, Gallagher L, Manoil C. 2017. Comprehensive Arrayed Transposon Mutant Library of Klebsiella pneumoniae Outbreak Strain KPNIH1. J Bacteriol 199.
