# Supplementary material for: Ethanolamine metabolism through two genetically distinct loci enables Klebsiella pneumoniae to bypass nutritional competition in the gut
Source: PLoS Pathog. 2024 May 7;20(5):e1012189. doi: 10.1371/journal.ppat.1012189 (PMC11101070; doi:10.1371/journal.ppat.1012189)
Supplement: S1 Data — (DOCX) [file ppat.1012189.s011.docx]

## S1 Data

## Genomic analysis to identify the presence of *eut* loci and their conservation across *K. pneumoniae species complex*

Genomes were obtained from 29 *Kp*SC studies (**S3 Table**). Studies reporting outbreaks or highly specific sampling strategies such as isolates selected for carriage of specific resistance genes etc. were excluded to reduce sampling bias. Studies without appropriate curated metadata including isolation source, country and year of isolation were also excluded. Reads were acquired from European Nucleotide Archive using the enaDataGet and enaGroupGet scripts from enaBrowserTools version 1.6. Reads were trimmed with Trim Galore version 0.5.0 using -q 20 option(1) then assembled with Unicycler version 0.4.7 with the --keep 0 option (2). The Graphical Fragment Assembly (GFA) files were then analysed for the number of GFA dead ends per assembly using getUnicyclerGraphStats_GFA.py from Unicycler. Assemblies with >200 GFA dead ends were excluded from the study. Assemblies were then analysed with Kleborate version 2.0.4 (3) to identify species and Sequence Types (STs). For genome annotation, Bakta version 1.1.1 (4) was used to annotate all genomes, followed by PopPUNK version 2.4.0 (5) to finely cluster isolates prior to dereplication. Dereplication was performed to reduce overrepresented samples within the dataset. Isolates which shared the same combination of ‘dbscan popPUNK cluster’, ‘source of isolation’ and ‘country of isolation’ were sorted, then the highest quality assemblies were selected using Assembly-Dereplicator version 0.1.0 (6). Phylogenetic analysis was performed using Panaroo version 1.2.8 and IQ-TREE 2 version 2.0.4 (7, 8).

*eut* genes and regulator binding sites were identified from the *K. pneumoniae* NTUH-K2044 genome (accession: GCF_000009885.1), then blastn (Blast+ version 2.9.0) (9) and filtered on 80% query coverage and identity. To identify regulator binding sites (EutR, NtrC and RpoN), the entire upstream region of each operon was identified as above, and then the sequence was screened via a nested blastn, and filtered on 70% identity. The distributions were then analyzed via the Twilight protocol (10).To analyze the genetic context of the long *eut* operon, the surrounding genomic context was extracted from genbanks using the slice_multi_genbank.py (<https://gist.github.com/bananabenana/20ff257f237d5a6e6f449fd7066577a1>). Clinker version 0.0.23 (11) was used for visualization.

**References**

1. Krueger F. 2015. Trim Galore: A wrapper tool around Cutadapt and FastQC to consistently apply quality and adapter trimming to FastQ files, with some extra functionality for MspI-digested RRBS-type (Reduced Representation Bisufite-Seq) libraries.

2. Wick RR, Judd LM, Gorrie CL, Holt KE. 2017. Unicycler: Resolving bacterial genome assemblies from short and long sequencing reads. PLoS Comput Biol 13:e1005595.

3. Lam MMC, Wick RR, Watts SC, Cerdeira LT, Wyres KL, Holt KE. 2021. A genomic surveillance framework and genotyping tool for Klebsiella pneumoniae and its related species complex. Nat Commun 12:4188.

4. Schwengers O, Jelonek L, Dieckmann MA, Beyvers S, Blom J, Goesmann A. 2021. Bakta: rapid and standardized annotation of bacterial genomes via alignment-free sequence identification. Microb Genom 7.

5. Lees JA, Harris SR, Tonkin-Hill G, Gladstone RA, Lo SW, Weiser JN, Corander J, Bentley SD, Croucher NJ. 2019. Fast and flexible bacterial genomic epidemiology with PopPUNK. Genome Res 29:304-316.

6. Wick RR, Holt KE. 2019. Benchmarking of long-read assemblers for prokaryote whole genome sequencing. F1000Res 8:2138.

7. Minh BQ, Schmidt HA, Chernomor O, Schrempf D, Woodhams MD, von Haeseler A, Lanfear R. 2020. IQ-TREE 2: New Models and Efficient Methods for Phylogenetic Inference in the Genomic Era. Mol Biol Evol 37:1530-1534.

8. Tonkin-Hill G, MacAlasdair N, Ruis C, Weimann A, Horesh G, Lees JA, Gladstone RA, Lo S, Beaudoin C, Floto RA, Frost SDW, Corander J, Bentley SD, Parkhill J. 2020. Producing polished prokaryotic pangenomes with the Panaroo pipeline. Genome Biol 21:180.

9. Camacho C, Coulouris G, Avagyan V, Ma N, Papadopoulos J, Bealer K, Madden TL. 2009. BLAST+: architecture and applications. BMC Bioinformatics 10:421.

10. Horesh G, Taylor-Brown A, McGimpsey S, Lassalle F, Corander J, Heinz E, Thomson NR. 2021. Different evolutionary trends form the twilight zone of the bacterial pan-genome. Microb Genom 7.

11. Schmidt BM, Davidson NM, Hawkins ADK, Bartolo R, Majewski IJ, Ekert PG, Oshlack A. 2018. Clinker: visualizing fusion genes detected in RNA-seq data. Gigascience 7.
